# Ultrasound-Activated Nanoplatform Counteracts Triple-Negative Breast Cancer via Remodeling Intratumoral Microbiota–Metabolism and Inducing Ferroptosis

**DOI:** 10.34133/bmr.0317

**Published:** 2026-02-03

**Authors:** Shuao Li, Yuxiu Gao, Danni Jiang, Xiaoyu Wu, Yanan Feng, Fang Chen, Ningning He, Shangyong Li, Luxia Jing, Cheng Zhao

**Affiliations:** ^1^ Department of Abdominal Ultrasound, The Affiliated Hospital of Qingdao University, Qingdao, Shandong 266003, China.; ^2^ School of Basic Medicine, Qingdao Medical College, Qingdao University, Qingdao 266071, China.

## Abstract

Triple-negative breast cancer (TNBC) remains therapeutically challenging owing to the paucity of broadly effective molecular targets. Piezoelectric nanomaterials that generate localized electric fields and reactive oxygen species under ultrasound (US) stimulation have emerged as a promising strategy for TNBC therapy. Here, we developed a US-activatable nanoplatform (HN-T/BT@Lip) in which toyocamycin-loaded CaCO_3_–carboxymethyl chitosan hybrid nanoparticles (HNs) and barium titanate (BaTiO_3_, BT) are co-encapsulated in folate-modified liposomes. US-activated HN-T/BT@Lip suppressed tumor growth and induced ferroptosis. Integrated transcriptomic, metabolomic, and microbiota profiling further revealed that this treatment disrupts glutathione metabolism, enhances susceptibility to lipid peroxidation, and perturbs iron homeostasis. These effects were closely associated with shifts in microbial community composition and altered levels of microbiota-derived metabolites. In vitro assays further demonstrated that the microbiota-associated metabolite trimethylamine *N*-oxide synergistically amplified lipid peroxidation under HN-T/BT@Lip + US treatment. Collectively, our findings demonstrate that US-activated HN-T/BT@Lip elicits potent ferroptosis in TNBC while concomitantly reshaping the intratumoral microbiota–metabolism landscape, and they highlight microbiota-derived metabolites such as trimethylamine *N*-oxide as potential modulators and biomarkers of nanotherapeutic ferroptosis.

## Introduction

Triple-negative breast cancer (TNBC) is the most aggressive subtype of breast cancer, accounting for approximately 15% to 20% of cases [1]. Because TNBC lacks expression of the estrogen receptor, the progesterone receptor, and human epidermal growth factor receptor 2, most patients do not benefit from receptor-directed therapies [2]. Surgery combined with neoadjuvant chemotherapy remains the standard of care for early-stage disease [3], whereas systemic chemotherapy is still the primary option for advanced or unresectable disease. Nevertheless, high recurrence rates and pronounced chemoresistance limit long-term efficacy [4], underscoring an urgent need for alternative therapeutic strategies.

In recent years, ultrasound (US)-activated piezoelectric materials, exemplified by barium titanate (BaTiO_3_, BT), have garnered increasing attention owing to their capacity to generate localized endogenous electric fields and reactive oxygen species (ROS) [5,6]. Unlike traditional tumor-treating fields, which disrupt mitosis by applying alternating and uniform external electric fields [7], piezoelectric materials generate highly localized surface potentials upon US stimulation [8], thereby enabling the precise modulation of diverse cellular signaling pathways, with calcium signaling being a prominent example. The concept of electrical stimulation induced by US-activated piezoelectric materials to regulate calcium signaling was originally explored in the fields of neuroscience and regenerative medicine [9]. For example, BT/reduced graphene oxide composite piezoelectric nanorods have been reported to activate voltage-gated calcium channels (VGCCs), thereby promoting neural differentiation and supporting repair after traumatic brain injury [10]. More recently, this mechanism has been extended to oncology, where US-activated piezoelectric nanomaterials have been reported to induce calcium overload through VGCC-mediated Ca^2+^ influx, along with synergistic ROS generation, thereby enhancing antitumor efficacy [11]. Collectively, these advances highlight the promise of piezoelectric materials as calcium-responsive actuators that can be integrated into drug-delivery systems for cross-disciplinary biomedical applications.

Despite these advances, a critical limitation persists: most current studies have primarily focused on the direct cytotoxic effects of piezoelectric materials on tumor cells, while their systemic biological mechanisms remain poorly understood [12]. In particular, the electric fields and ROS generated by the piezoelectric effect can also modulate microbial physiology [13], raising the possibility that a US-activated piezoelectric nanoplatform may reprogram the intratumoral microbiota and its metabolic outputs. These microbiota-level alterations may shape tumor progression and modulate treatment response. Consistent with this view, accumulating evidence indicates that intratumoral microbiota participate in metabolic reprogramming, for instance, by promoting GLUT1-mediated lactate accumulation in oral squamous cell carcinoma and enhancing glycolysis via the ENO1-IT1 pathway in colorectal cancer [14,15]. They also appear to influence treatment response, as intratumoral *Lactobacillus reuteri*-derived metabolites and microbiota-produced inosine have been reported to augment responses to immune checkpoint inhibitors [16,17].

**Fig. 1.**
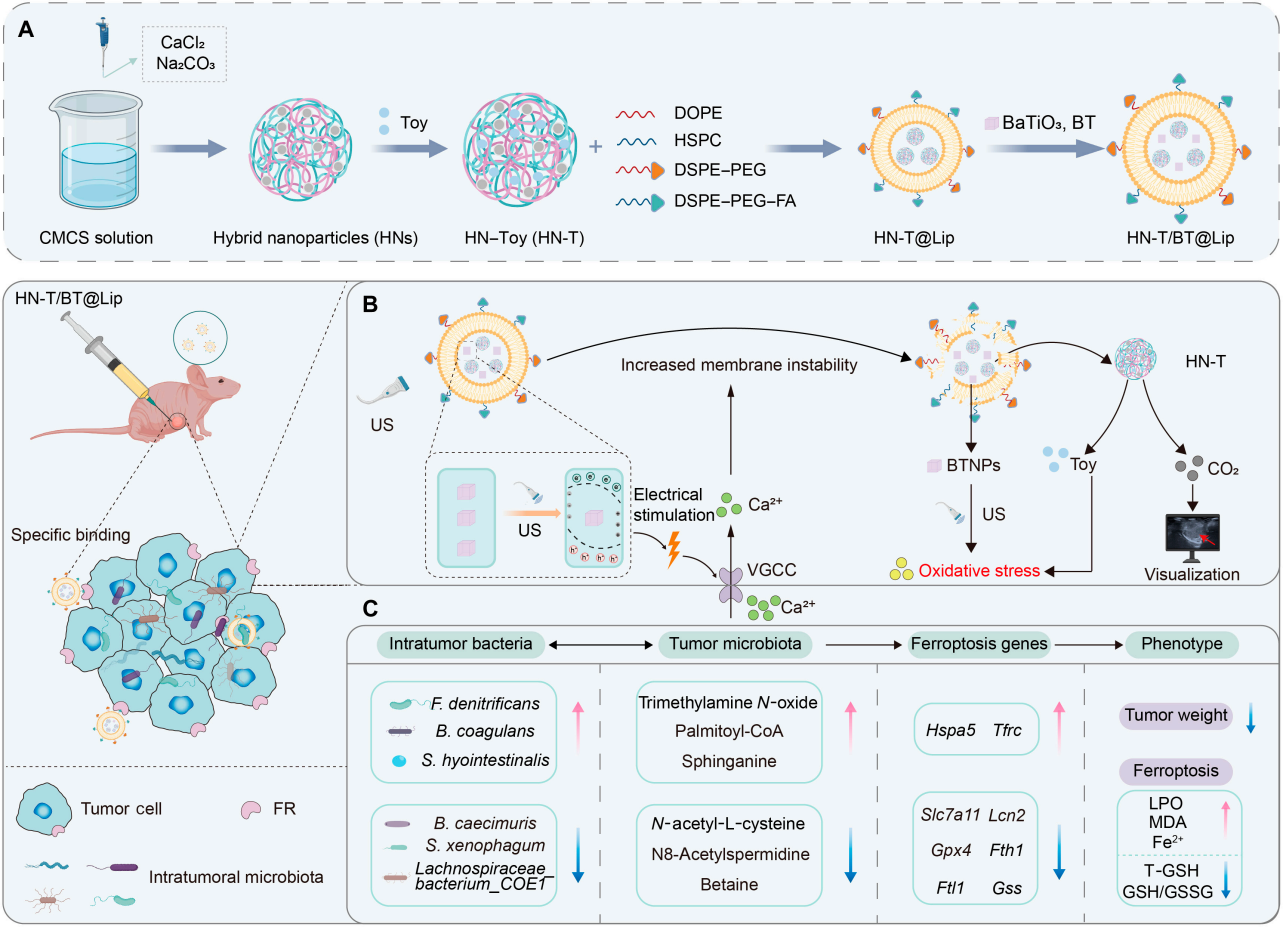
(A) Schematic diagram of HN-T/BT@Lip preparation. (B) Schematic illustration of the ultrasound (US)-triggered piezoelectric response of HN-T/BT@Lip. (C) HN-T/BT@Lip reshapes intratumoral microbiota and metabolism to sensitize triple-negative breast cancer (TNBC) cells to ferroptosis (created with BioRender.com). HN, carbonate–carboxymethyl chitosan hybrid nanoparticle; BT, barium titanate (BaTiO_3_); CMCS, carboxymethyl chitosan; Toy, toyocamycin; DOPE, 1,2-dioleoyl-*sn*-glycero-3-phosphoethanolamine; HSPC, hydrogenated soy phosphatidylcholine; DSPE–PEG, 1,2-distearoyl-*sn*-glycero-3-phosphoethanolamine–polyethylene glycol; DSPE–PEG–FA, folic acid-conjugated DSPE–PEG; VGCC, voltage-gated calcium channel; BTNPs, barium titanate nanoparticles; FR, folate receptor; LPO, lipid peroxidation; MDA, malondialdehyde; T-GSH, total glutathione; GSH, glutathione; GSSG, oxidized glutathione.

In this study, we constructed HN-T/BT@Lip, a nanoplatform activated by US stimulation and calcium signaling. The nanoparticle core comprises calcium carbonate–carboxymethyl chitosan (CaCO_3_–CMCS) hybrid nanoparticles (HNs) loaded with toyocamycin (Toy), an endoplasmic reticulum stress inducer that cooperates with piezoelectric activation to amplify oxidative stress [18]. This nanoparticle core is encapsulated within folate-modified liposomes co-loaded with BT, enabling targeted delivery and US-triggered piezoelectric activation (Fig. 1). Upon US activation, the platform generates localized electric fields, induces intracellular Ca^2+^ influx, and destabilizes the liposomal membrane [19], thereby facilitating nanoparticle core release. Under an acidic tumor microenvironment, CaCO_3_ within the HN core decomposes, thereby accelerating Toy release and simultaneously generating CO_2_ microbubbles, which act as efficient acoustic scatterers to enhance US imaging contrast and enable real-time visualization [20]. Through integrated multi-omics analyses and functional validation, we show that US-activated HN-T/BT@Lip induces ferroptosis, remodels intratumoral microbiota and metabolic pathways, and perturbs redox homeostasis in TNBC. These findings underscore the potential of nanoplatforms to modulate the interplay among intratumoral microbiota, metabolism, and cell death, providing mechanistic insights to guide therapeutic innovation in TNBC and inform the rational design of piezoelectric nanoplatforms.

## Materials and Methods

### Chemicals and reagents

CMCS was purchased from Yuanye Bio-Technology Co., Ltd. (Shanghai, China). According to the certificate of analysis, CMCS exhibited a degree of substitution of 85.8%, a viscosity of 36.84 mPa·s, and an average molecular weight ranging from 10 to 20 kDa. Calcium chloride (CaCl_2_), sodium carbonate (Na_2_CO_3_), Toy, 1,2-dioleoyl-*sn*-glycero-3-phosphoethanolamine (DOPE), and hydrogenated soy phosphatidylcholine (HSPC) were purchased from Macklin Biochemical Co., Ltd. (Shanghai, China). 1,2-Distearoyl-*sn*-glycero-3-phosphoethanolamine–*N*-[methoxy(polyethylene glycol)-2000] (DSPE–PEG_2000_) and folic acid-conjugated DSPE–PEG_2000_ (DSPE–PEG–FA) were purchased from Yusi Pharmaceutical Technology Co., Ltd. (Shanghai, China). BT (BaTiO_3_) was purchased from XFNANO Materials Tech Co., Ltd. (Nanjing, China). Trimethylamine *N*-oxide (TMAO) was purchased from Sigma-Aldrich (Shanghai, China). Cell Counting Kit-8 (CCK-8), calcein-AM/propidium iodide (PI) Live/Dead Cell Viability Assay Kit, and an ROS detection kit (2′,7′-dichlorodihydrofluorescein diacetate [DCFH-DA]) were purchased from Beyotime Biotechnology (Shanghai, China). A malondialdehyde (MDA) assay kit, a total glutathione (T-GSH)/oxidized glutathione (GSSG) assay kit, a ferrous ion (Fe^2+^) assay kit (colorimetric), and a lipid peroxidation (LPO) kit (BODIPY 581/591 C11) were obtained from Wuhan Servicebio Technology Co., Ltd. (Wuhan, China). Unless otherwise stated, all reagents were of analytical grade and used without further purification.

### Preparation of HN-T/BT@Lip

#### Synthesis of HN and Toy loading (HN-T)

Calcium carbonate–CMCS HNs were prepared as follows: CMCS (10 mg) was dissolved in deionized water, and 20 mM CaCl_2_ was added under continuous magnetic stirring at room temperature for 1 h. Subsequently, an equal volume of 20 mM Na_2_CO_3_ was added and the mixture was stirred for an additional 30 min. The suspension was centrifuged to remove unreacted components. The pellet was resuspended in water and lyophilized overnight.

For Toy loading, Toy (3 mg) and HNs (6 mg) were co-dispersed in 10 ml of deionized water and stirred at 22 to 25 °C for 24 h. The dispersion was centrifuged, and the pellet was washed 3 times with deionized water. All supernatants were collected for quantification. The Toy concentration in the supernatant was determined by ultraviolet–visible spectrophotometry at 279 nm, using deionized water as the blank (Lambda 750s, PE Instruments, USA). The encapsulation efficiency (EE) and loading capacity (LC) were calculated as

$$\mathrm{EE}\%=\frac{m_{1}-m_{2}\;}{m_{0}}\times 100\;\left(\%\right)$$ (1)

$$\mathrm{LC}\%=\frac{m_{1}-m_{2}}{m_{1}}\times 100\;\left(\%\right)$$ (2)

where *m*_1_ is the initial Toy mass, *m*_2_ is the Toy mass remaining in the supernatant (unencapsulated), and *m*_0_ is the total mass of HN-T nanoparticles.

#### Assembly of BT@Lip and HN-T/BT@Lip

HSPC (4 mg), DOPE (4 mg), cholesterol (1 mg), DSPE–PEG–FA (2 mg), and DSPE–PEG_2000_ (2 mg) were dissolved in chloroform and evaporated at 50 °C for 15 min in a rotary evaporator to form a thin film. The film was hydrated with 10 ml of HN-T dispersion, followed by bath sonication for 5 min. The suspension was incubated at 37 °C for 1 h, cooled to 25 °C, and probe-sonicated in an ice bath for 2 min to obtain HN-T@Lip.

BT (20 mg) and DSPE–PEG_2000_ (24 mg) were co-dissolved in tetrahydrofuran and bath-sonicated to ensure uniform dispersion. The organic phase was slowly injected into 10 ml of distilled water under continuous sonication and further sonicated for 5 min. The resulting dispersion was purified by 3 cycles of centrifugation with deionized water to remove residual lipids and solvent. The purified BT dispersion was mixed at a 1:1 volume ratio with preformed HN-T@Lip and extruded 10 times through a polycarbonate membrane using a mini-extruder to obtain the final HN-T/BT@Lip formulation. Blank liposomes (Blank-Lip) were prepared using the same lipid composition and procedures as HN-T/BT@Lip, except that neither HN-T nanoparticles nor BT was added. BT@Lip was prepared following the same procedure as HN-T/BT@Lip, except that Blank-Lip was used instead of HN-T@Lip during the extrusion step. The detailed preparation workflow was thoroughly described in our previous study [21].

### Characterization of nanoparticles

Transmission electron microscopy (TEM; JEM-2100, JEOL Ltd., Tokyo, Japan) was employed to examine the morphology of HN-T, BT@Lip, and HN-T/BT@Lip. Samples were prepared by dispersing nanoparticles in deionized water, depositing the dispersion onto carbon-coated copper grids, and air-drying prior to imaging. The hydrodynamic diameter and zeta potential were determined at 25 °C using Zetasizer Nano ZS (Malvern Instruments, Malvern, UK). Measurements were performed on freshly prepared dispersions to evaluate colloidal stability and surface charge. X-ray diffraction (XRD) patterns were recorded using an x-ray diffractometer system (SmartLab SE; Rigaku, Tokyo, Japan). Fourier transform infrared (FTIR) spectra were collected on a Nicolet iS20 spectrometer (Thermo Fisher Scientific, Waltham, MA, USA) to identify characteristic stretching vibrations of functional groups.

### Drug release behavior

Two distinct drug release mechanisms were designed to evaluate the drug release behavior of the system: (a) pH-responsive release and (b) US/calcium-triggered release.

#### pH-responsive release

HN-T nanoparticles were loaded into presoaked dialysis bags, which were then placed in 150 ml of 2-(*N*-morpholino)ethanesulfonic acid (MES)-buffered saline (pH 7.4 or 5.5) containing 1% fetal bovine serum. The dialysis system was maintained at 37 °C under gentle orbital shaking to ensure uniform dispersion and consistent diffusion dynamics. At designated time points (0.5, 1, 2, 4, 6, 12, 24, and 48 h), 5-ml aliquots were collected from the release medium and replenished with an equal volume of fresh buffer immediately to maintain sink conditions. The concentration of released Toy was determined using an ultraviolet–visible spectrophotometer (*λ* = 279 nm; Thermo Fisher Scientific, Wilmington, DE, USA), using deionized water as the blank.

#### US/calcium-triggered release

Three DOPE-enriched lipid formulations with low (5 mg; 30%), medium (6 mg; 40%), and high (7 mg; 50%) DOPE contents were incubated in MES-buffered saline (pH 5.5) containing 5 mM CaCl_2_. US stimulation was administered at 0, 2, and 4 h using a therapeutic US apparatus (DM-300K; DeMai Ultrasound, China) operating at 1 MHz and 1 W cm^−2^ for 3 min. Drug release and Toy quantification were performed as described in the “pH-responsive release” section.

### In vitro and in vivo US imaging of HN-T/BT@Lip

#### In vitro imaging

Lyophilized HN-T/BT@Lip samples were reconstituted in MES-buffered saline (pH 7.4 or 5.5) and loaded into the fingertip sections of latex gloves to serve as imaging phantoms. These phantoms were immersed in a 37 °C water bath to mimic physiological temperature conditions. The US contrast-enhancing capability of the nanoparticles was assessed using a small-animal US imaging system (VINNO 6 LAB; VINNO Technology, Suzhou, China).

To further evaluate concentration-dependent visualization, HN-T/BT@Lip dispersions (0, 25, 50, 100, and 200 μg/ml) in MES-buffered saline (pH 5.5) were loaded into individual glove phantoms under identical conditions. B-mode US images were acquired using consistent imaging parameters, and regions of interest were manually delineated within each sample area. The mean gray values were quantified using the ImageJ software and plotted to assess the dose-dependent enhancement in echogenicity.

#### In vivo imaging

Tumor-bearing BALB/c nude mice were anesthetized with isoflurane gas (induction at 3% to 4% and maintenance at 1.5% to 2%). Guided by US, 150 μl of the HN-T/BT@Lip formulation was intratumorally injected. Piezoelectric activation was initiated using the therapeutic US apparatus described above. US imaging of the tumor site was conducted using a small-animal US imaging system (VINNO 6 LAB; VINNO Technology, Suzhou, China).

### Cell culture and in vitro assays

4T1 cells and human umbilical vein endothelial cells (HUVECs) were cultured under standard conditions at 37 °C in a humidified atmosphere containing 5% CO_2_ and subcultured routinely before reaching 80% to 90% confluency.

In vitro assays included cytotoxicity evaluation of HN-T/BT@Lip and BT@Lip, live/dead cell staining, and intracellular ROS detection, which were performed according to standard protocols. Fluorescence imaging was performed using a conventional fluorescence microscope.

### In vitro cytotoxicity assay

In vitro cytotoxicity was evaluated using CCK-8 in accordance with the manufacturer’s instructions. HUVECs and 4T1 cells were seeded into 96-well plates at a density of 5 × 10^3^ cells per well and allowed to adhere overnight. Cells were then treated with HN-T/BT@Lip at various concentrations (0, 25, 50, 100, 200, and 400 μg/ml) and further incubated for 24 or 48 h.

To assess US-enhanced cytotoxicity, 4T1 cells were exposed to different formulations with or without US exposure prior to incubation. US treatment was applied at 1.0 MHz with an acoustic intensity of 1.5 W cm^−2^, a 50% duty cycle, and a duration of 3 min. Following treatment, 10 μl of CCK-8 solution was added to each well, and the plates were incubated for an additional 2 h at 37 °C. The absorbance at 450 nm was measured using a microplate reader (iMark; BIO-RAD, Hercules, CA, USA) to determine cell viability.

### Calcein-AM and PI dual staining for live/dead cell imaging

A calcein-AM/PI dual-staining assay was performed to assess cell viability and visualize treatment-induced cytotoxicity in 4T1 cells. Briefly, cells were seeded into glass-bottom confocal dishes at a density of 1 × 10^5^ cells per well and incubated at 37 °C for 24 h to allow attachment. The cells were then subjected to different treatments, including control, free Toy, BT-Lip + US, HN-T/BT@Lip, and HN-T/BT@Lip + US irradiation (1.0 MHz, 1.5 W cm^−2^, 50% duty cycle, 3 min).

Following treatment, cells were washed with phosphate-buffered saline (PBS) and stained with calcein-AM (2 μM) and PI (4 μM) in accordance with the manufacturer’s instructions. The stained cells were incubated at 37 °C for 20 min in the dark to prevent photobleaching. Fluorescence images were captured using an inverted fluorescence microscope (Nikon, Tokyo, Japan), where live cells exhibited green fluorescence and dead cells appeared red. Quantitative analysis of cell viability and mean fluorescence intensity was conducted using the ImageJ software.

### In vitro ROS generation assay

The intracellular ROS levels in 4T1 cells were evaluated using the fluorescent probe DCFH-DA (Beyotime, Shanghai, China). Cells were seeded into glass-bottom confocal culture dishes at a density of 1 × 10^5^ cells per well and incubated at 37 °C for 24 h to allow attachment. Following attachment, cells were treated with various formulations, including control, free Toy, BT-Lip + US, HN-T/BT@Lip, and HN-T/BT@Lip + US irradiation (US device: WED-100 therapeutic US system, 1.0 MHz, 1.5 W cm^−2^, 50% duty cycle, 3 min).

After treatment, cells were incubated with 10 μM DCFH-DA in serum-free medium at 37 °C for 30 min in the dark. Excess probe was removed by washing 3 times with serum-free medium, followed by final rinses with PBS. Fluorescence images were then acquired using an inverted fluorescence microscope (Nikon, Tokyo, Japan), and ROS levels were quantified based on green fluorescence intensity using the ImageJ software.

### In vivo antitumor effects

All experimental procedures were approved by the Committee on the Ethics of Animal Experiments of the Medical College of Qingdao University (QDU-AEC-2024781). All animal housing and experiments were conducted in strict accordance with institutional guidelines for the care and use of laboratory animals.

Healthy female BALB/c nude mice (4 to 5 weeks old) were purchased from Jereh Biotech Co., Ltd. (Qingdao, China) and maintained under specific-pathogen-free conditions. All animal experiments were approved in advance by the Animal Care and Use Committee of Qingdao University.

To establish subcutaneous tumor models, 4T1 cells (1 × 10^7^ cells/ml, 0.1 ml) were injected into the right axilla of each mouse after disinfection of the injection site. When the tumor volume reached approximately 200 mm^3^, mice were randomly assigned to 6 groups (*n* = 6 per group): (a) control, (b) Blank-Lip, (c) free Toy, (d) BT@Lip + US (US; 1.0 W cm^−2^, 1.0 MHz, 50% duty cycle, 5 min), (e) HN-T/BT@Lip, and (f) HN-T/BT@Lip + US. Nanoparticles (BT@Lip or HN-T/BT@Lip) were administered via intratumoral injection.

Treatments were administered on days 1, 3, 5, and 7. During the 15-d therapeutic period, tumor volume and body weight were measured every other day. Tumor volume was calculated as *V* = 1/2 × length × (width)^2^. At the end of the study, mice were euthanized, and tumors along with major organs (heart, liver, spleen, lung, and kidney) were harvested. Tumor weights were recorded individually, and tissues were subjected to hematoxylin and eosin (H&E) staining for histological analysis.

### Histopathological staining

Tumor tissues and major organs (heart, liver, spleen, lung, and kidney) were fixed in 4% paraformaldehyde for 24 h, embedded in paraffin, and sectioned at 5 μm. Following deparaffinization, sections were subjected to H&E staining. The stained sections were examined using a light microscope (Olympus, Tokyo, Japan) at magnifications of ×40 and ×200.

### 16S rRNA sequencing

Fresh tumor tissue samples were collected under sterile conditions and stored in cryogenic tubes at −80 °C until analysis. Total genomic DNA was extracted, quantified, and assessed for integrity using 1% agarose gel electrophoresis. The V3 to V4 hypervariable regions of the 16S ribosomal RNA (rRNA) gene were amplified by polymerase chain reaction (PCR) using primers 341F (5′-CCTAYGGGRBGCASCAG-3′) and 806R (5′-GGACTACNNGGGTATCTAAT-3′). The purified and quantified PCR products were subsequently used to construct sequencing libraries.

For 16S rRNA sequencing, an average sequencing depth of approximately 50,000 tags per sample was achieved. Data processing involved stringent quality control procedures: raw reads were demultiplexed according to barcode sequences, with barcodes and primers removed; paired-end reads (R1 and R2) were merged using FLASH; and low-quality reads (>10% bases with a Phred quality score <20) and chimeric sequences were filtered out to obtain high-quality effective tags. Amplicon sequence variants (ASVs) were generated using the DADA2 algorithm. Taxonomic classification of ASVs was performed using the classify-sklearn method in QIIME2, with a pre-trained naive Bayes classifier (97% sequence similarity) based on the SILVA 138.1 reference database. Species abundance profiles were generated from phylum to species levels according to ASV annotations and sample feature tables.

### Transcriptome analysis

Approximately 150 mg of tumor tissue was homogenized, and total RNA was extracted using the Sparkjade RNA Extraction Kit (no. AC0202, Sparkjade) in accordance with the manufacturer’s instructions. RNA integrity and concentration were assessed using Agilent 2100 Bioanalyzer (Agilent Technologies, Santa Clara, CA, USA). Complementary DNA libraries were subsequently constructed, and their quality and purity were evaluated using both Agilent 2100 DNA Bioanalyzer and Quant-iT PicoGreen dsDNA Assay Kit (Thermo Fisher Scientific, Waltham, MA, USA).

High-throughput sequencing was performed on an Illumina Genome Analyzer platform (Berry Genomics Co., Ltd., Beijing, China). Differentially expressed genes (DEGs) were identified based on the criteria of |log_2_FC| > 1 and *P* < 0.05. Volcano plots were generated using the R software (version 4.5.0) to visualize DEGs. Functional annotation and pathway enrichment analyses were conducted using the Kyoto Encyclopedia of Genes and Genomes (KEGG) database (https://www.genome.jp/kegg/) and the Gene Ontology (GO) database (http://geneontology.org/).

### Untargeted metabolomics analysis

Tumor tissue (≈100 mg) was homogenized in 1 ml of methanol:acetonitrile:water (2:2:1, v/v) and sonicated and then centrifuged to precipitate proteins. The cleared extract was analyzed on a ultrahigh-performance liquid chromatography system (Ultimate 3000, Thermo Fisher Scientific) fitted with an ACQUITY UPLC HSS T3 column (1.8 μm, 2.1 × 100 mm; Waters) and coupled to a Q Exactive hybrid quadrupole–Orbitrap mass spectrometer (Thermo Fisher Scientific). Data were collected in positive- and negative-ion modes using full mass spectrometry (MS) and data-dependent MS, with acetonitrile (A) and 0.1% formic acid (B) as the mobile phases. Detailed procedures were performed as described in our previous study [22].

### Western blotting analysis

Western blotting was conducted to evaluate protein expression. Approximately 100 mg of tumor tissue was lysed in pre-chilled radioimmunoprecipitation assay buffer (Beyotime Biotechnology, Shanghai, China) supplemented with protease and phosphatase inhibitors. The lysates were centrifuged, and the supernatants were collected for protein quantification using BCA Protein Assay Kit (Beyotime Biotechnology). Equal amounts of protein were mixed with 5× loading buffer, boiled at 100 °C for 10 min, separated by 10% sodium dodecyl sulfate–polyacrylamide gel electrophoresis, and transferred onto polyvinylidene difluoride membranes.

After blocking, the membranes were incubated overnight at 4 °C with a primary antibody against GPX4 (glutathione peroxidase 4) (rabbit polyclonal, 1:1,000, Servicebio, GB115275, Wuhan, China), followed by an additional 1-h incubation at room temperature with horseradish peroxidase-conjugated goat anti-rabbit immunoglobulin G secondary antibody (1:3,000, Servicebio, GB23303, Wuhan, China). Protein bands were visualized using an enhanced chemiluminescence detection kit (Beyotime Biotechnology) and quantified using the ImageJ software.

### Detection of LPO

LPO in 4T1 cells was assessed using the fluorescent probe BODIPY 581/591 C11 (Servicebio, Wuhan, China). Briefly, cells were seeded into 6-well plates at a density sufficient to reach approximately 80% confluence after overnight incubation at 37 °C in a humidified atmosphere containing 5% CO_2_. Following the indicated treatments, cells were stained in accordance with the manufacturer’s instructions. After incubation with the probe, cells were gently washed with PBS to remove excess dye, and fluorescence images were acquired using a fluorescence microscope (Nikon, Tokyo, Japan). LPO was qualitatively evaluated based on a fluorescence shift from red to green, indicative of the oxidative degradation of polyunsaturated lipids.

### Detection of MDA

The MDA levels in tumor tissues were quantified using a commercial assay kit (G4302; Servicebio, Wuhan, China) in accordance with the manufacturer’s protocol. Freshly excised tumor tissue was homogenized in buffer at a ratio of 1:9 (w/v) and centrifuged at 10,000 × g for 10 min at 4 °C. The supernatant was mixed with MDA working solution, incubated in a 95 °C water bath for 40 min, cooled to room temperature, and centrifuged again. The absorbance of the supernatant was measured at 532 nm, and MDA concentrations were calculated from a standard curve.

### Detection of Fe^2+^

The Fe^2+^ content in tumor tissues was determined using a ferrous ion assay kit (G4323; Servicebio, Wuhan, China) according to the manufacturer’s instructions. Briefly, tumor tissues were weighed and homogenized with extraction buffer at a weight-to-volume ratio of 1:9 (g/ml) in an ice–water bath to prepare a 10% tissue homogenate. The homogenates were centrifuged at 10,000 × g for 10 min at 4 °C, and the supernatants were collected for analysis. Reaction mixtures were incubated at 37 °C for 40 min and centrifuged at 10,000 × g for 5 min. Absorbance was measured at 593 nm, and Fe^2+^ concentrations were calculated based on a standard curve.

### Detection of T-GSH and GSSG

T-GSH and GSSG levels in tumor tissues were measured using a glutathione (GSH) assay kit (G4304; Servicebio, Wuhan, China) following the manufacturer’s protocol. Fresh tumor tissues were homogenized in protein removal reagent at a ratio of 1:9 (w/v) and centrifuged at 10,000 × g for 10 to 15 min at 4 °C. For T-GSH measurement, supernatants were mixed with GSH detection working solution and incubated at room temperature, followed by substrate addition, and further incubated at 25 °C for 20 min. Absorbance was recorded at 412 nm. For GSSG measurement, samples were pretreated with a GSH scavenger to eliminate reduced GSH and then analyzed using the same detection procedure. T-GSH and GSSG concentrations were calculated from standard curves, and reduced GSH levels were determined as T-GSH − 2 × GSSG.

### Statistical analysis

Statistical analyses were conducted using GraphPad Prism (version 10.1.2) and R software (version 4.5.0). Data are presented as mean ± standard error of the mean, with 3 to 6 biological replicates per experimental group. The Shapiro–Wilk test was applied to assess data normality. For normally distributed data (*P* > 0.05), 2-tailed unpaired Student *t* tests were applied for pairwise comparisons, and one-way analysis of variance followed by Tukey’s post hoc test was used for multiple-group comparisons. When the assumptions of normality or homogeneity of variance were not met, the Wilcoxon rank-sum test was employed for 2-group comparisons. Statistical significance was defined as *P* < 0.05. Significance levels were denoted as *P < 0.05, **P < 0.01, ***P < 0.001, ****P < 0.0001, and “ns”, indicating no significant difference.

## Results

### Preparation and characterization of HN-T/BT@Lip

**Fig. 2.**
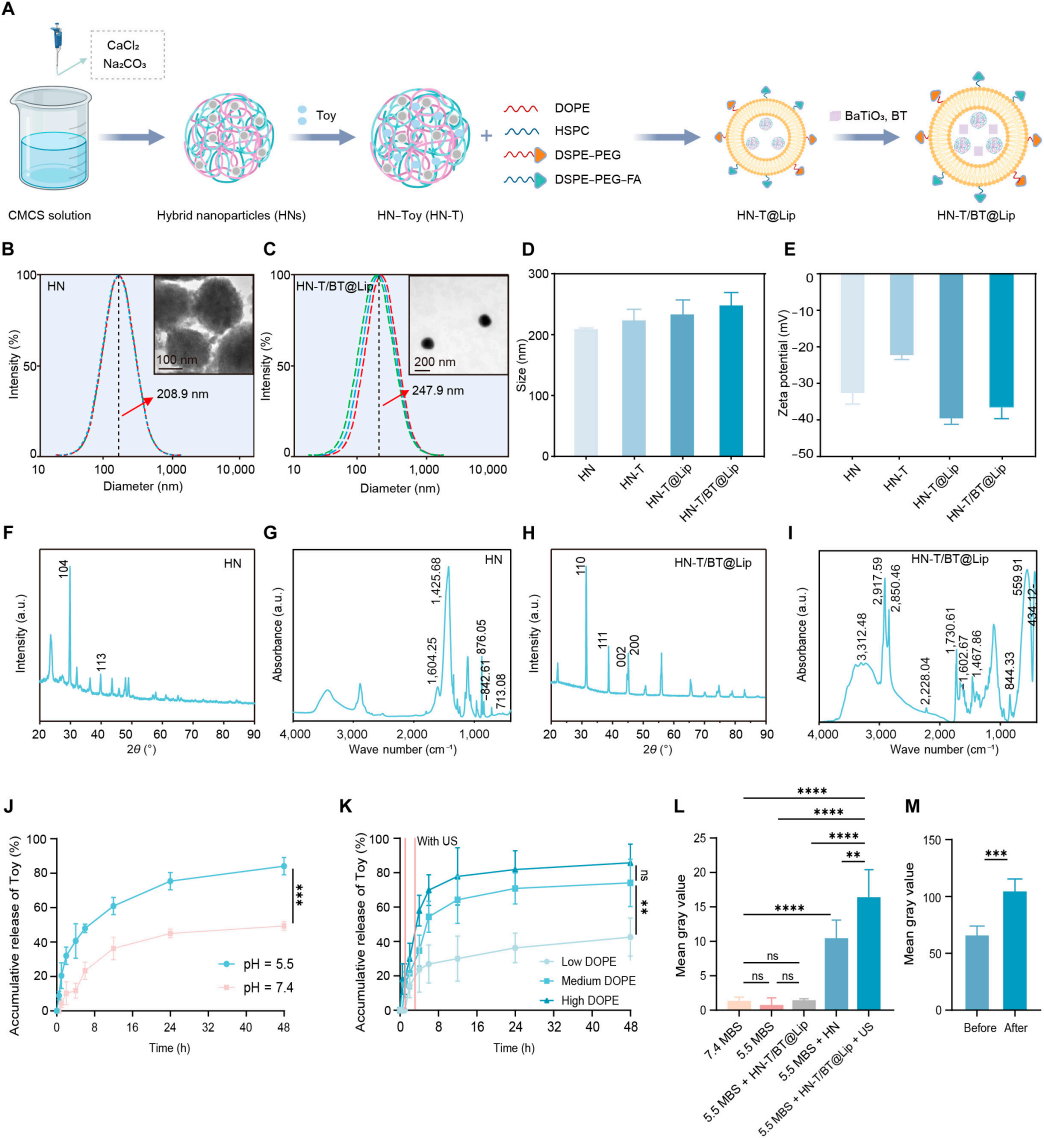
Preparation and characterization of HN-T/BT@Lip. (A) Schematic illustration of the preparation of HN-T/BT@Lip. (B) Transmission electron microscopy (TEM) of HN-T (scale bar, 100 nm). (C) TEM of HN-T/BT@Lip (scale bar, 200 nm). (D) Hydrodynamic diameters of HN, HN-T, HN-T@Lip, and HN-T/BT@Lip measured by dynamic light scattering (DLS) (*n* = 3). (E) Zeta potentials of the corresponding formulations (*n* = 3). (F and G) X-ray diffraction (XRD) and Fourier transform infrared (FTIR) spectra of HN-T. (H and I) XRD and FTIR spectra of HN-T/BT@Lip. (J) Cumulative Toy release profiles at pH 7.4 and 5.5 (*n* = 5). (K) US-triggered release under 5 mM CaCl_2_ conditions with different DOPE contents; US: 1.0 MHz, 1.0 W cm^−2^, 50% duty cycle, 3 min (*n* = 5). (L) In vitro US B-mode imaging in phantoms at pH 7.4 and 5.5 under different formulations; signal quantified as mean gray value within region of interest (ROI) (*n* = 5). MBS, 2-(*N*-morpholino)ethanesulfonic acid (MES)-buffered saline. (M) In vivo US imaging before and after intratumoral injection of HN-T/BT@Lip and US treatment; signal quantified as mean gray value (*n* = 5). **P < 0.01; ***P < 0.001; ****P < 0.0001.

A stepwise assembly strategy was employed to construct HN-T/BT@Lip (Fig. 2A). Initially, acid-degradable HNs were prepared, followed by Toy loading to yield HN-T. The drug-loaded cores were subsequently co-encapsulated with BT within DOPE-enriched liposomes, producing HN-T/BT@Lip that enables drug delivery, acid-triggered release, and US-triggered piezoelectric activation.

TEM revealed that the HNs were spherical and uniform in morphology (Fig. 2B). Dynamic light scattering measured a hydrodynamic diameter of 208.9 ± 0.99 nm, and electrophoretic light scattering showed a zeta potential of −32.59 ± 1.77 mV, indicative of good colloidal stability (Fig. 2D and E). Following Toy loading, the hydrodynamic diameter increased to 223.1 ± 10.9 nm and the zeta potential became less negative (approximately −22.0 mV), consistent with electrostatic interactions between Toy and CaCO_3_ that reduce the density of surface –COO^−^/–OH^−^ groups. The XRD patterns of HNs showed characteristic peaks at around 29.4° and 39.4° (2*θ*), confirming the formation of the calcite phase of CaCO_3_ [23]. These features suggest a thermodynamically stable crystalline structure (Fig. 2F) that is expected to maintain integrity at physiological pH yet dissolve under acidic pH, enabling pH-responsive degradation. FTIR spectra further validated the co-assembly of CMCS and CaCO_3_, as evidenced by the asymmetric/symmetric stretching of COO^−^ (1,604.25 and 1,425.68 cm^−1^) and carbonate bending vibrations (876.05, 842.61, and 713.08 cm^−1^) (Fig. 2G). BT@Lip, a BaTiO_3_-loaded liposomal formulation extensively studied in our previous work, was also morphologically characterized in the present study. TEM revealed a well-defined core–shell morphology (Fig. S1), thereby corroborating its structural stability as a constituent of the overall HN-T/BT@Lip formulation.

The final formulation, HN-T/BT@Lip, exhibited a vesicular morphology as observed via TEM (Fig. 2C), with a hydrodynamic diameter of 247.9 ± 12.3 nm and a negatively charged surface (Fig. 2D and E). The successful incorporation of BT was confirmed by distinct XRD peaks at around 31.5°, 38.9°, 45.2°, and 50.8° (2*θ*), consistent with the tetragonal BT phase (Fig. 2H) [24]. The FTIR spectrum of HN-T/BT@Lip exhibited distinct vibrational features corresponding to each component, including O–H/N–H stretching (3,312.48 cm^−1^), lipid-associated C–H stretching (2,917.59 and 2,850.46 cm^−1^), Toy’s C≡N stretching (2,228.04 cm^−1^), ester C=O stretching (1,730.61 cm^−1^), CMCS-associated COO^−^ and N–H bands (1,602.67 and 1,425.68 cm^−1^), carbonate bending (844.33 cm^−1^), and Ti–O vibrations (599.91 and 434.12 cm^−1^) from BT (Fig. 2I).

Drug release studies showed higher release at pH 5.5 than at neutral pH, with cumulative release exceeding 80% at 48 h, consistent with acid-triggered CaCO_3_ dissolution (Fig. 2J). To evaluate the combined effects of Ca^2+^ and US on liposomal membrane destabilization and to optimize US-triggered release, we examined liposomes with varying DOPE contents in MES-buffered saline containing 5 mM CaCl_2_. A higher DOPE content correlated with faster US-triggered drug release (Fig. 2K). Additionally, the HNs imparted intrinsic US imaging capability to the nanoplatform. Under mildly acidic conditions that mimic the tumor microenvironment, CaCO_3_ decomposes to generate CO_2_ bubbles. These CO_2_ bubbles act as strong acoustic scatterers, which induce pronounced backscattering of incident US waves and consequently return greater acoustic energy to the transducer. The enhanced backscattering elevates local echo intensity and improves imaging contrast, thereby enabling clearer and more sensitive real-time US visualization. As shown in Fig. 2L and Fig. S2A, compared with the control group (pH 7.4), both the HN group and the HN-T/BT@Lip + US group showed markedly increased echo intensities at pH 5.5. Furthermore, in the HN-T/BT@Lip + US group, the brightness and mean gray values of US images increased progressively as the nanoparticle concentration increased (Fig. S2B and C). Consistently, after intratumoral injection of HN-T/BT@Lip, the tumor region showed a pronounced increase in US signal relative to baseline, and quantitative grayscale analysis further confirmed this enhancement (Fig. 2M and Fig. S2D). Based on formulation screening (Tables S1 and S2) using EE and LC as primary criteria, the optimized composition was Toy:HN = 1:2 (w/w) and DOPE:HSPC = 1:1 (w/w) for subsequent investigations.

### In vitro cytocompatibility and US-activated antitumor activity of HN-T/BT@Lip

**Fig. 3.**
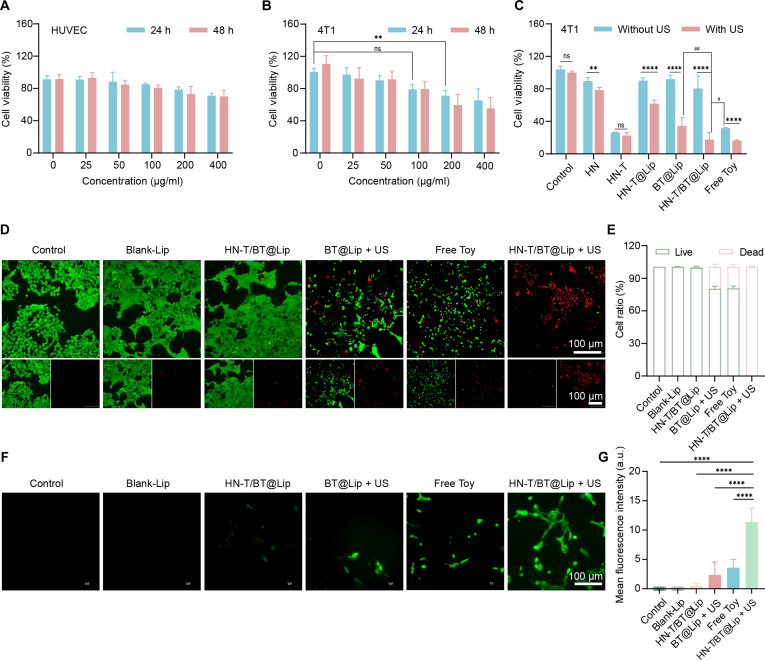
Evaluation of in vitro cytocompatibility and US-activated antitumor activity. (A) Viability of human umbilical vein endothelial cells (HUVECs) treated with HN-T/BT@Lip (0 to 400 μg/ml) for 24 and 48 h (*n* = 4). (B) Viability of 4T1 cells under the same conditions (*n* = 4). (C) Viability of 4T1 cells after different treatments with or without US activation (1.0 MHz, 1.0 W/cm^2^, 50% duty cycle, 3 min, *n* = 4). (D) Live/dead staining of 4T1 cells (green: calcein-AM; red: propidium iodide [PI]; scale bar, 100 μm). (E) Quantification of live-cell ratio using ImageJ (*n* = 5). (F) Intracellular reactive oxygen species (ROS) detected by 2′,7′-dichlorodihydrofluorescein diacetate (DCFH-DA) staining (bar, 100 μm). (G) Quantification of ROS intensity per ROI using ImageJ (*n* = 5). **P < 0.01 and ****P < 0.0001, ^#^*P* < 0.05 and ^##^*P* < 0.01, for selected intergroup comparisons as indicated (C).

To evaluate the basal cytocompatibility of the designed nanoplatform, we first conducted cytotoxicity assays on HUVECs and 4T1 cells in the absence of US stimulation (Fig. 3A and B). The results indicated minimal cytotoxicity of HN-T/BT@Lip toward both cell lines across concentrations ranging from 0 to 400 μg/ml, confirming its inherent biocompatibility. Based on its negligible toxicity, a working concentration of 100 μg/ml was selected to ensure consistency in subsequent therapeutic evaluations. In our prior work, BT@Lip was also evaluated for cytotoxicity, and at a working concentration of 100 μg/ml, it did not significantly reduce cell viability (Fig. S3).

The therapeutic potential of the US-responsive design was subsequently evaluated to determine its activation efficiency. As shown in Fig. 3C, HN-T/BT@Lip alone exhibited limited effect on 4T1 viability, whereas HN-T/BT@Lip + US significantly reduced cell viability. This outcome notably outperformed both the BT@Lip + US and free Toy groups, underscoring the superiority of the piezoelectric nanoplatform in enhancing tumor cell killing.

Consistent with the viability assay, live/dead staining (Fig. 3D) showed the highest PI-positive (red) fraction in the HN-T/BT@Lip + US group, indicating substantial membrane disruption. Quantitative image analysis corroborated a significant reduction in viability relative to all other groups (Fig. 3E).

To further probe the mechanism of enhanced cytotoxicity, intracellular ROS levels were evaluated via DCFH-DA staining. As shown in Fig. 3F and G, the HN-T/BT@Lip + US group displayed the highest green fluorescence, consistent with elevated intracellular oxidative stress. Compared to those of the free Toy, BT@Lip + US, and untreated control groups, ROS levels were significantly higher, supporting a role for oxidative stress in the observed cell death.

Collectively, these results indicate that HN-T/BT@Lip exhibits US-activated antitumor activity while maintaining in vitro cytocompatibility, supporting subsequent mechanistic studies of cell-death pathways and preclinical therapeutic evaluation.

### In vivo antitumor efficacy of HN-T/BT@Lip

**Fig. 4.**
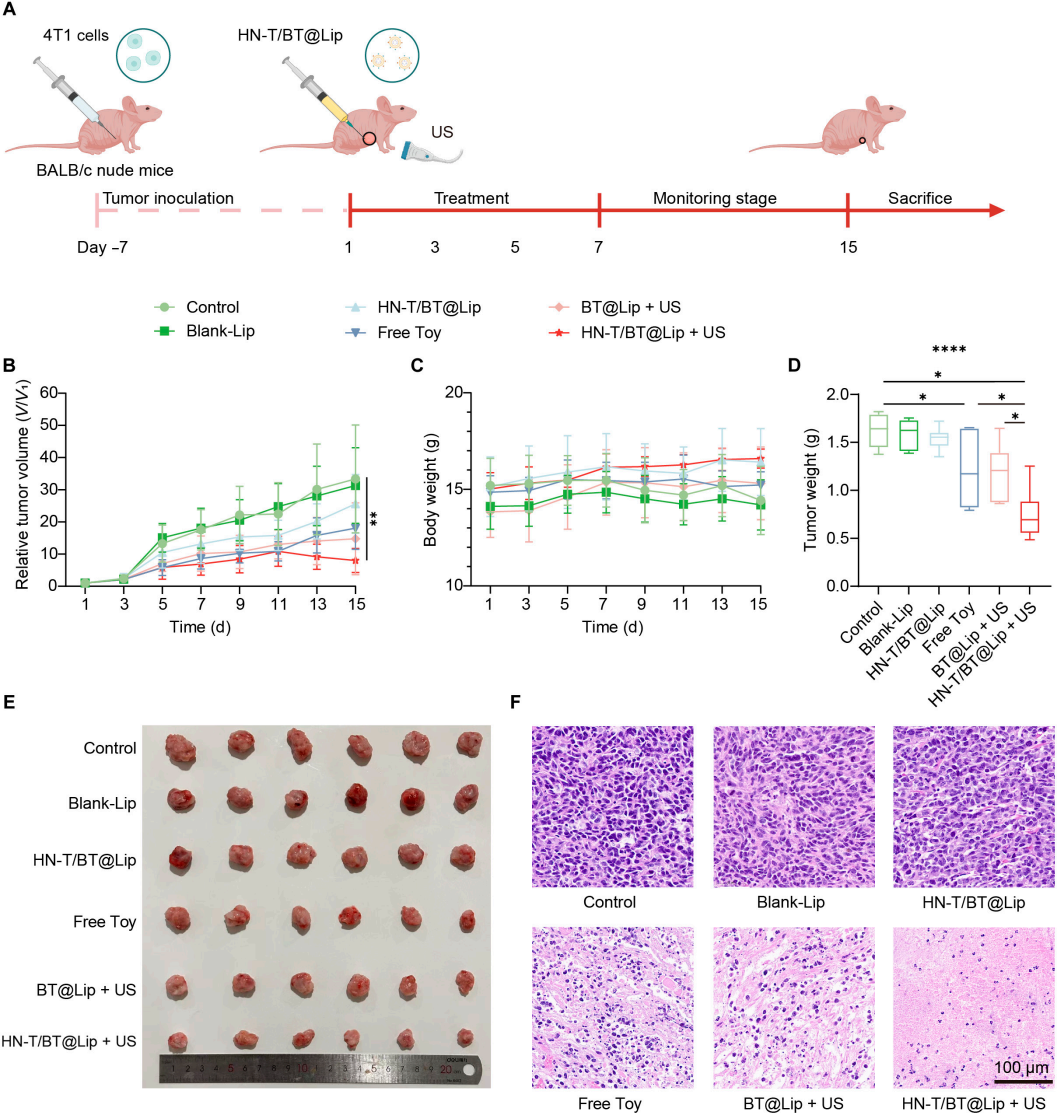
In vivo antitumor efficacy of HN-T/BT@Lip. (A) Treatment scheme for 4T1 tumor-bearing BALB/c nude mice. (B) Tumor growth curves (*V*/*V*_1_), (C) body-weight changes during treatment, (D) terminal tumor weight of each mouse, and (E) representative photographs of excised tumors at the endpoint (*n* = 6). (F) Hematoxylin and eosin (H&E) staining of tumor sections (scale bar, 100 μm). *P < 0.05; **P < 0.01; ****P < 0.0001.

Given the excellent in vitro biocompatibility and US-activated antitumor efficacy of HN-T/BT@Lip, we further evaluated its in vivo therapeutic efficacy using a 4T1 subcutaneous xenograft model in BALB/c nude mice. As illustrated in Fig. 4A, 4T1 cells were inoculated into the left axillary region of each mouse to establish tumors. Once tumor volumes reached approximately 100 mm^3^, mice were randomized into treatment groups. Local administration was performed, followed by US activation as appropriate. Tumor growth was monitored throughout the 15-d treatment period, after which tumors and major organs were collected for histological and multi-omics analyses.

As shown in Fig. 4B, the tumor growth in the HN-T/BT@Lip + US group was significantly inhibited, indicating strong therapeutic efficacy. Tumor weight measurements (Fig. 4D) further confirmed the contributions of drug-mediated cytotoxicity and piezoelectric activation in suppressing tumor progression, with the combination therapy eliciting a markedly synergistic antitumor effect. Consistently, tumor inhibition rate (TIR) analysis (Fig. S4) revealed that HN-T/BT@Lip + US achieved a TIR of 54.10%, substantially higher than those of Toy alone (25.39%) and BT@Lip + US (26.99%), underscoring the therapeutic advantage conferred by the integrated nanoplatform. Representative tumor photographs (Fig. 4E) were qualitatively consistent with these measurements, illustrating reduced tumor burden with HN-T/BT@Lip + US. No significant changes in body weight were observed in all groups during treatment (Fig. 4C), suggesting that the therapeutic regimen was well tolerated and exhibited no signs of systemic toxicity.

To assess the therapeutic effects at the tissue level, H&E staining was performed on resected tumor sections (Fig. 4F). The HN-T/BT@Lip + US group exhibited extensive tissue necrosis, pyknotic nuclei, and severe architectural disruption. In contrast, moderate histopathological alterations were observed in the Toy and BT@Lip + US groups, while the control and Blank-Lip groups retained largely intact tumor architecture.

In addition, H&E staining of major organs, including the heart, liver, spleen, lung, and kidney, revealed no obvious inflammatory infiltration, necrosis, or structural abnormalities in any treatment group (Fig. S5), which, together with the stable body weight, suggests that the therapeutic regimen did not induce apparent systemic toxicity. Collectively, these findings indicate that HN-T/BT@Lip, when activated by US, not only induces tumor cell damage in vitro and in vivo but also exhibits a favorable systemic safety profile, providing a robust basis for subsequent mechanistic investigations.

### Transcriptomic alterations after HN-T/BT@Lip + US

To investigate mechanisms underlying the antitumor effects observed in vivo following HN-T/BT@Lip + US treatment, RNA sequencing was conducted on freshly excised tumor tissues. Principal component analysis (Fig. S6) showed a distinct segregation between the HN-T/BT@Lip + US and control groups, indicating substantial transcriptional differences.

**Fig. 5.**
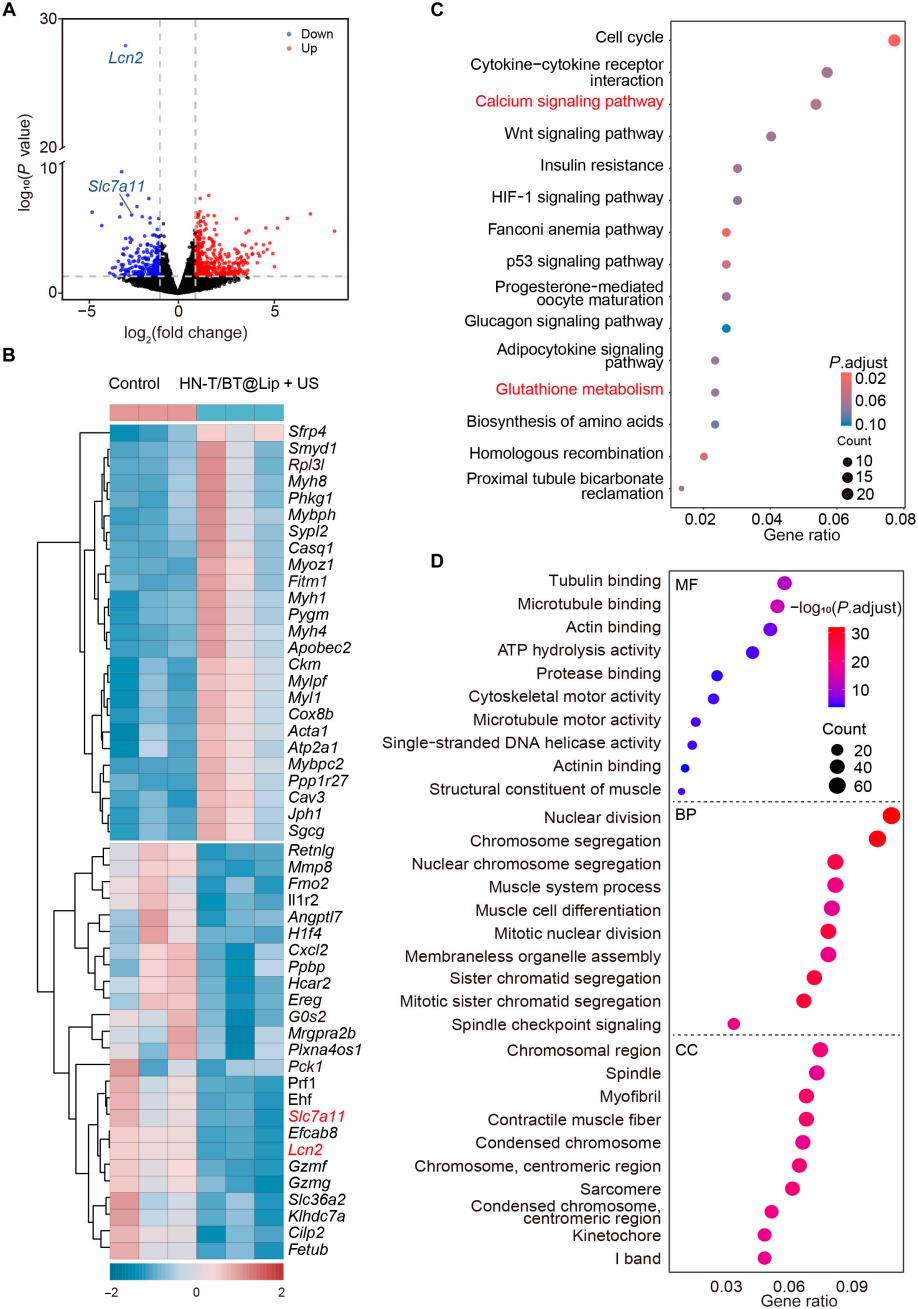
Transcriptomic alterations of HN-T/BT@Lip + US treatment. (A) Volcano plot of differentially expressed genes (DEGs) between control and HN-T/BT@Lip + US tumors. Red represents upregulated genes, and blue indicates down-regulated genes. (B) Hierarchical clustering heatmap of DEGs between the control and HN-T/BT@Lip + US groups. (C) Kyoto Encyclopedia of Genes and Genomes (KEGG) pathway enrichment analysis. (D) Gene Ontology (GO) enrichment analysis of DEGs indicating key biological processes, molecular functions, and cellular components affected by the treatment. MF, Molecular Function; BP, Biological Process; CC, Cellular Component.

Using thresholds of |log_2_FC| > 1 and *P* < 0.05, 676 DEGs were identified, including 234 upregulated and 442 down-regulated transcripts (Fig. 5A). To visualize these changes, the top 25 upregulated and top 25 down-regulated DEGs were presented in a heatmap (Fig. 5B), alongside a volcano plot for comprehensive overview. Notably, 2 ferroptosis-related genes, *Lcn2* and *Slc7a11*, were significantly down-regulated in the HN-T/BT@Lip + US group relative to those in controls. *Lcn2* has been implicated in limiting ferroptosis by modulating iron handling and antioxidant defenses [25], while *Slc7a11*, the light-chain subunit of the system Xc^−^ transporter, is essential for cystine uptake and GSH biosynthesis [26]. The concurrent down-regulation of these genes suggests that ferroptosis may play a crucial role in the therapeutic mechanism.

To further elucidate the functional implications of the DEGs, KEGG pathway enrichment analysis was performed (Fig. 5C), revealing significant enrichment in GSH metabolism, cell cycle, calcium signaling pathway, and cytokine–cytokine receptor interaction [27]. These findings imply that the treatment may elicit antitumor efficacy by disrupting redox balance, suppressing cellular proliferation, and modulating immune–inflammatory responses. GO enrichment analysis (Fig. 5D) demonstrated significant enrichment of mitosis-related terms across all 3 GO domains, including nuclear division (Biological Process), microtubule motor activity (Molecular Function), and kinetochore (Cellular Component). Overall, transcriptomic data indicate that the treatment engages multiple pathways, and the concomitant down-regulation of *Slc7a11* and *Lcn2* supports a role for ferroptosis in its mechanism of action.

### Metabolic remodeling induced by HN-T/BT@Lip + US

**Fig. 6.**
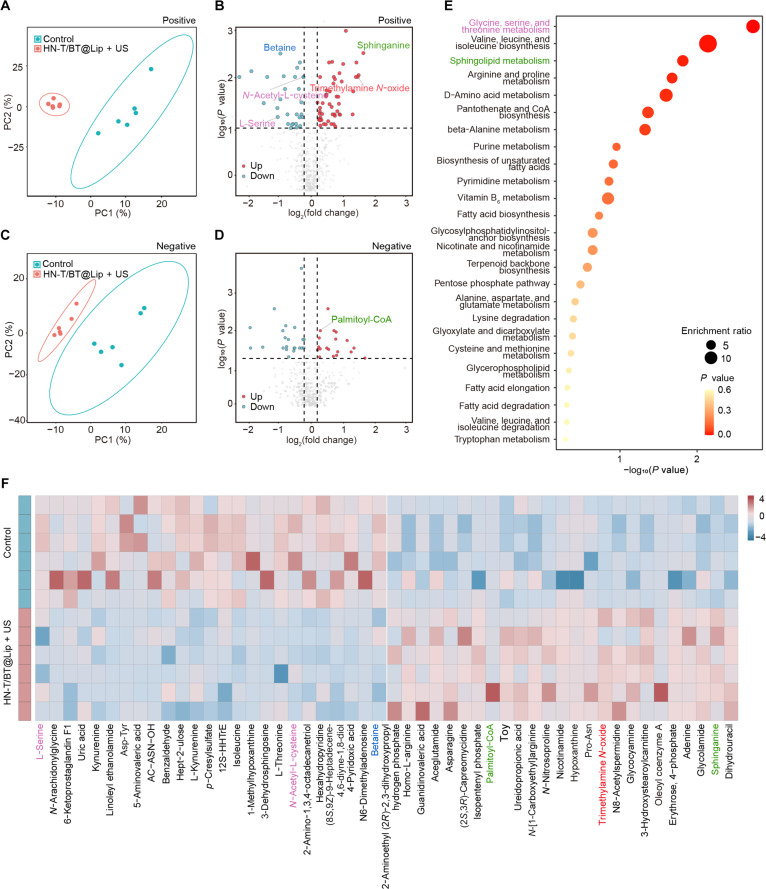
Metabolomic remodeling induced by HN-T/BT@Lip + US. Partial least squares discriminant analysis (PLS-DA) analysis of tumor tissues in positive- (A) and negative-ion modes (C). Volcano plots illustrating differential metabolites in positive (B) and negative (D) modes. (E) KEGG pathway enrichment highlighting glycine/serine/threonine metabolism and sphingolipid metabolism. (F) Heatmap showing differential metabolites between the control and HN-T/BT@Lip + US groups.

Given that ferroptosis is a metabolism-linked form of regulated cell death [28], we investigated whether the transcriptomic changes were accompanied by metabolic reprogramming. We performed untargeted metabolomic profiling via liquid chromatography–mass spectrometry (LC–MS) on tumor tissues from both the treatment and control groups. Partial least squares discriminant analysis showed clear group separation between HN-T/BT@Lip + US and controls in both ion modes, suggesting significant treatment-induced metabolic reprogramming (Fig. 6A and C). Using filtering thresholds of |log_2_FC| > 0.2 and *P* < 0.05, a total of 121 significantly altered metabolites were identified, including 51 upregulated and 29 down-regulated in positive-ion mode and 19 upregulated and 22 down-regulated in negative-ion mode (Fig. 6B and D). KEGG pathway enrichment (Fig. 6E) and heatmap analysis (Fig. 6F) jointly highlighted 2 ferroptosis-relevant metabolic axes that showed consistent alteration trends: (a) Sphingolipid axis: Levels of palmitoyl-CoA and its downstream metabolite sphinganine were significantly elevated, corresponding to enrichment in the sphingolipid metabolism pathway. These alterations suggest disruptions in lipid homeostasis and redox regulation. Notably, sphinganine is directly implicated in ferroptosis-related pathways, and its marked upregulation underscores the activation of LPO cascades [29,30]. (b) GSH axis: Precursors involved in GSH biosynthesis, such as *N*-acetyl-l-cysteine and l-serine, were substantially depleted, consistent with the enrichment of the glycine/serine/threonine metabolism pathway. This depletion pattern indicates compromised antioxidant capacity, which may heighten cellular vulnerability to oxidative damage [31]. Furthermore, TMAO, a host–microbiota co-metabolite reported to sensitize cells to ferroptosis, was elevated, supporting a link between intratumoral microbiota alterations and a ferroptosis-associated metabolic phenotype [32].

**Fig. 7.**
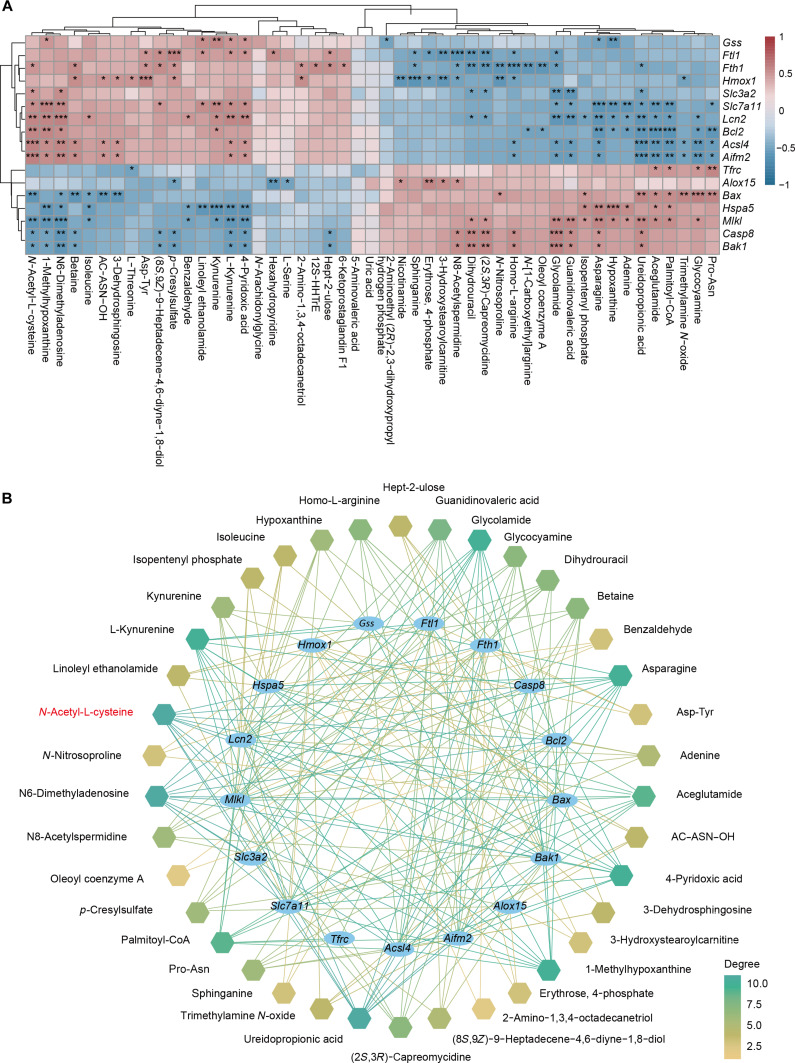
Metabolite–gene correlation analysis. (A) Heatmap showing correlations between key ferroptosis-related genes and differential metabolites. (B) Correlation network of ferroptosis-related genes (inner circles) and metabolites (outer circles), with color intensity indicating correlation strength. *P < 0.05; **P < 0.01; ***P < 0.001.

To further clarify the underlying mechanisms, we performed Spearman correlation analysis between differentially expressed ferroptosis-related genes and the differential metabolites. As shown in the heatmap (Fig. 7A), *N*-acetylcysteine (NAC) exhibited positive correlations with several genes involved in antioxidant defense and iron regulation within the GSH metabolism axis, including *Slc7a11*, *Lcn2*, *Fth1*, and *Slc3a2*, but showed negative correlations with genes associated with cell death and inflammation, such as *Bax*, *Bak1*, *Casp8*, and *Mlkl*. The ferroptosis gene–metabolite correlation network further underscores the central role of NAC (Fig. 7B). With respect to LPO and iron homeostasis, sphinganine showed inverse correlations with *Hmox1*, *Fth1*, and *Ftl1*, consistent with reduced antioxidant/iron-buffering capacity and increased susceptibility to LPO. Furthermore, TMAO was positively correlated with Bax and negatively correlated with *Aifm2* and *Hmox1*, suggesting its involvement in amplifying oxidative stress. In summary, HN-T/BT@Lip + US reshapes the tumor metabolic landscape by synergistically promoting LPO and depleting GSH precursors, thereby enhancing ferroptosis susceptibility. This metabolic reprogramming provides mechanistic support for the platform’s antitumor efficacy. Notably, alterations in several host–microbiota co-metabolites suggest a potential contribution of the intratumoral microbiota to this process.

### Analysis of intratumoral microbiota after HN-T/BT@Lip + US

**Fig. 8.**
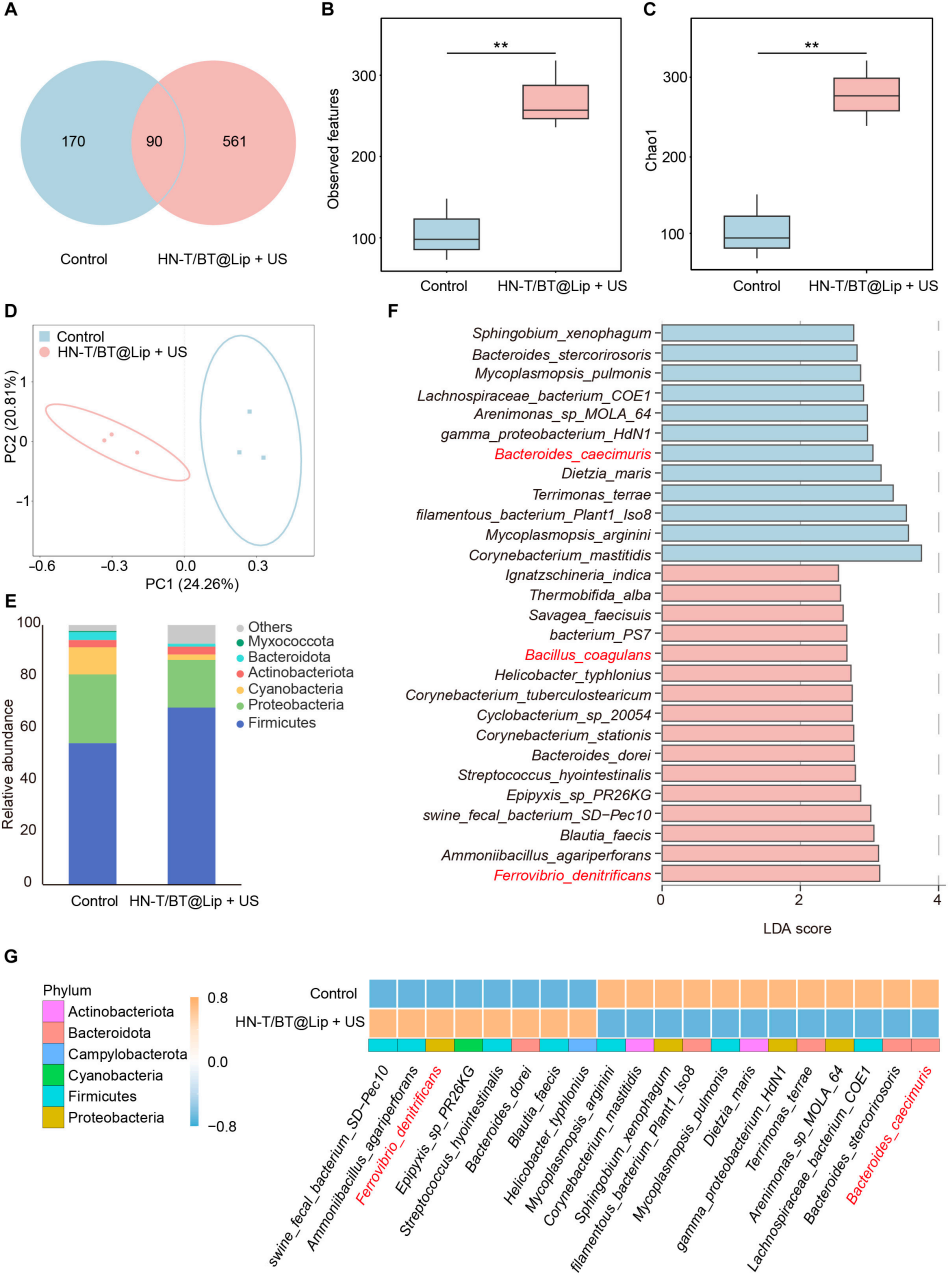
Analysis of intratumoral microbiota after HN-T/BT@Lip + US. (A) Venn diagram showing shared and unique amplicon sequence variants (ASVs) between the control and treatment groups. (B and C) α-diversity indicating higher richness after treatment. (D) Principal coordinate analysis (PCoA) of Jaccard distances showing group separation. (E) Phylum-level composition. (F) Linear discriminant analysis effect size (LEfSe) (linear discriminant analysis [LDA] score > 2.5) identifying taxa enriched after treatment. (G) Heatmap of discriminant taxa across group. **P < 0.01.

Motivated by evidence that piezoelectricity-generated electric fields and ROS can influence microbial physiology and by our observation of altered host–microbiota co-metabolites, we examined whether HN-T/BT@Lip + US modulates the composition of the intratumoral microbiota. To this end, 16S rRNA gene sequencing was performed on excised tumor tissues to systematically assess microbial compositional changes. After adapter trimming, quality filtering, and chimera removal, ASV analysis identified 170 control-specific and 561 treatment-specific ASVs, with 90 core ASVs shared between both groups (Fig. 8A). At the α-diversity level, the treatment group exhibited significantly higher observed features and Chao1 indices (Fig. 8B and C), indicating increased microbial richness, which may reflect microbial reprogramming induced by the treatment. Furthermore, principal coordinate analysis based on Jaccard distance showed clear separation between the 2 groups (Fig. 8D), suggesting that HN-T/BT@Lip + US altered the intratumoral microbial community structure. At the phylum level, the relative abundances of Firmicutes and Proteobacteria increased in the treatment group, while those of Bacteroidota and Actinobacteriota decreased (Fig. 8E).

To further identify key species contributing to this divergence, linear discriminant analysis (LDA) effect size (LDA score > 2.5) was utilized to screen for significantly enriched taxa (Fig. 8F). Heatmap visualization (Fig. 8G) illustrated that *Ferrovibrio denitrificans* and *Bacillus coagulans* were dominant in the treatment group, whereas *Bacteroides caecimuris* was predominant in the control group. According to prior studies, *F. denitrificans* has been linked to iron-associated metabolism and denitrification pathways [33]. Similarly, *B. coagulans* has been reported to promote ferroptosis by down-regulating the key ferroptosis suppressor GPX4 through its bacterial culture filtrate and by activating the Keap1/Nrf2 signaling pathway [34]. Collectively, these findings suggest that HN-T/BT@Lip + US may modulate ferroptosis sensitivity by weakening the local antioxidant barrier, while promoting the enrichment of iron metabolism-related taxa.

To explore potential associations between microbes and metabolites, we performed Spearman correlation analysis between the top 20 differentially abundant species and significantly altered metabolites (Fig. S7). *F. denitrificans*, enriched in the treatment group, exhibited positive correlations with TMAO, sphinganine, and glycocyamine and a negative correlation with betaine. Similarly, *B. coagulans* was positively associated with TMAO and sphinganine. Conversely, *B. caecimuris*, enriched in the control group, showed negative correlations with sphinganine and homo-l-arginine. Furthermore, these correlations underscore a strong association between intratumoral microbial alterations and metabolic remodeling.

### HN-T/BT@Lip + US activates ferroptosis through coordinated intratumoral microbiota–metabolic remodeling

**Fig. 9.**
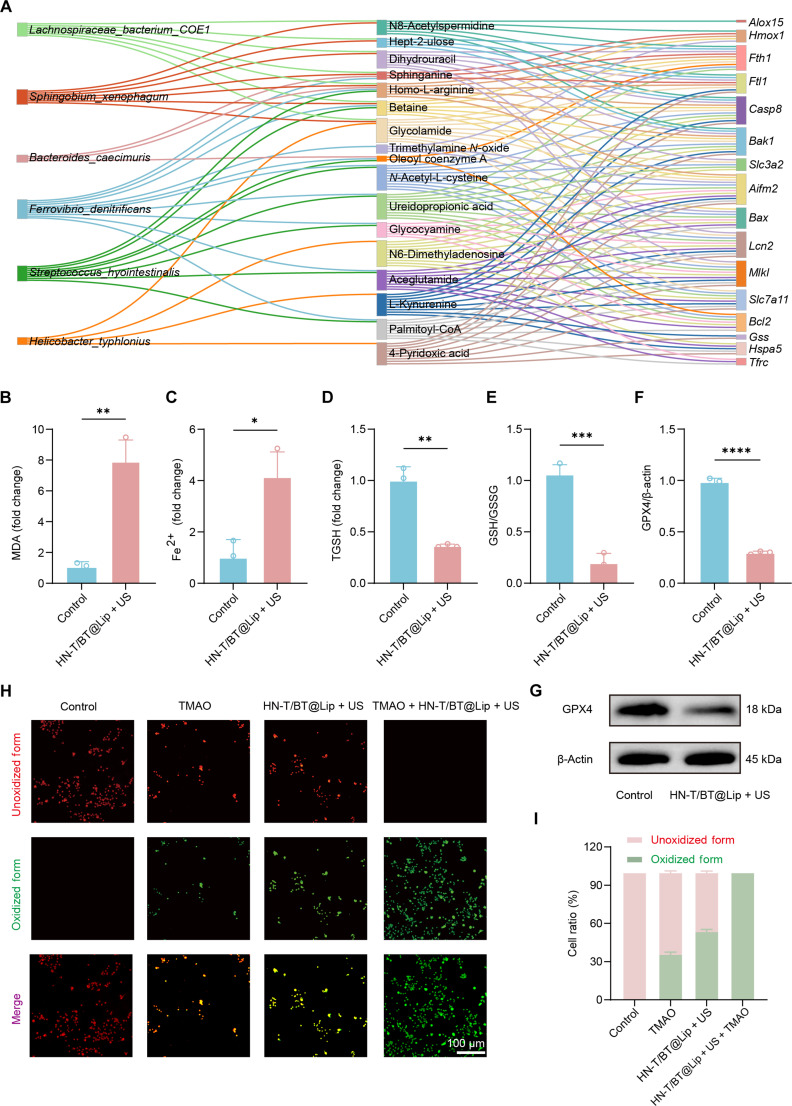
HN-T/BT@Lip + US activates ferroptosis through coordinated intratumoral microbiota–metabolic remodeling. (A) The Sankey diagram shows the interactions among intratumoral microbiota, core metabolites, and genes related to ferroptosis. (B to E) Tumor biochemistry showing increased MDA and Fe^2+^ and reduced total GSH and GSH:GSSG. (F and G) GPX4 (glutathione peroxidase 4) Western blots and densitometry of GPX4/β-actin; (H) C11-BODIPY imaging of cells under the indicated treatments (red, unoxidized; green, oxidized); (I) quantification of mean fluorescence. Trimethylamine *N*-oxide (TMAO): 400 mM. *P < 0.05; **P < 0.01; ***P < 0.001; ****P < 0.0001.

To elucidate system-level regulation of ferroptosis by the intratumoral microbiome, we integrated 16S rRNA, LC–MS metabolomic, and RNA sequencing transcriptomic datasets and computed 2-sided Spearman’s rank correlations (|*ρ*| > 0.6) to identify microbe–metabolite associations. These associations were subsequently integrated into a 3-layer Sankey diagram (Fig. 9A), with detailed procedures performed as described in our previous study [35]. The resulting network included 6 representative microbial taxa, 17 differential metabolites, and 17 ferroptosis-associated genes. *F. denitrificans* exhibited extensive correlations with various metabolites. Notably, *N*-acetyl-l-cysteine was closely associated with ferroptosis-related genes involved in the GSH axis (e.g., *Gss* and *Slc7a11*). Sphinganine was associated with *Hmox1*, *Fth1*, and *Ftl1*, and TMAO showed strong associations with *Bax*, *Aifm2*, and *Hmox1*, collectively highlighting a ferroptosis-regulatory module centered on iron homeostasis, antioxidant defense, and LPO dysregulation.

Therefore, we further validated the ferroptosis-inducing capacity of HN-T/BT@Lip + US at both tissue and cellular levels. In tumor tissues, key biochemical markers of ferroptosis showed important dysregulation [36]: MDA, a terminal product of LPO, was elevated (Fig. 9B); intracellular Fe^2+^ levels increased (Fig. 9C); T-GSH was reduced along with a reduced GSH/GSSG ratio (Fig. 9D and E), indicating impaired redox buffering. Moreover, the expression of GPX4, a central antiferroptotic enzyme, was markedly down-regulated (Fig. 9F and G). Collectively, these results demonstrate that HN-T/BT@Lip + US induces ferroptosis in TNBC via GSH depletion, GPX4 suppression, and LPO accumulation.

To further assess the role of intratumoral microbiota and its metabolites in this process, we conducted functional validation using TMAO as a representative co-metabolite. TMAO was significantly enriched in our metabolomic analysis and has been closely linked to microbiota–host metabolic interactions (Fig. S8). In C11-BODIPY 581/591 LPO assays, TMAO alone can increase membrane LPO. When combined with HN-T/BT@Lip under US activation, a further increase in LPO was observed (Fig. 9H and I), indicating a synergistic role of TMAO in promoting oxidative lipid damage. Collectively, evidence from tissue biochemistry, enzyme expression, and cellular imaging supports that HN-T/BT@Lip + US induces ferroptosis in TNBC by disrupting the balance between antioxidant defense and iron homeostasis. Furthermore, the synergistic effect of TMAO supports the hypothesis that this piezoelectric nanoplatform may modulate intratumoral microbiota–metabolism–oxidative stress interactions within the tumor microenvironment and potentially amplify ferroptosis.

## Discussion

In this study, we developed a piezoelectric nanoplatform (HN-T/BT@Lip) to help address the therapeutic challenges in TNBC by enabling on-demand drug release and precise therapeutic intervention. Physicochemical characterization confirmed that the platform possesses excellent colloidal stability and biocompatibility and exhibits robust antitumor efficacy both in vitro and in vivo. Through integrated multi-omics analyses and functional validation, HN-T/BT@Lip + US was shown to induce ferroptosis in TNBC. Moreover, we systematically demonstrated that this platform modulates microbial composition and metabolic pathways, increases oxidative stress, and weakens antioxidant defenses, thereby potentially enhancing ferroptotic sensitivity in TNBC. Further analysis showed that specific microbially derived metabolites (e.g., TMAO) markedly amplified LPO induced by HN-T/BT@Lip + US, supporting a role for microbiota–metabolite interactions in ferroptosis regulation. These findings not only deepen our understanding of microbial–metabolic cross talk in regulating ferroptosis but also provide a conceptual foundation for designing personalized nanotherapeutics that target the heterogeneous intratumoral microbiota.

Nanomedicine represents an emerging and promising strategy for cancer therapy [37]. Unlike conventional piezoelectric nanotherapeutic strategies that primarily focus on direct cytotoxicity [38,39], the HN-T/BT@Lip platform introduces dual innovations in both functional design and mechanistic action. This system enables multidimensional responsiveness to both the tumor microenvironment and external US, thereby achieving on-demand drug release and precise therapeutic intervention. Structurally, the CaCO_3_–CMCS hybrid core acts as a stable scaffold for Toy loading while also serving as a pH-responsive trigger, which enables rapid disintegration in the mildly acidic tumor microenvironment to accelerate drug release and generate CO_2_ bubbles that enhance US contrast for real-time imaging (Fig. 2J and Fig. S2). BT functions as the piezoelectric element, producing localized electric fields and promoting Ca^2+^ influx under US activation, thereby enhancing ROS generation and triggering subsequent signaling cascades. Meanwhile, the DOPE lipid component undergoes Ca^2+^-induced membrane dehydration and phase separation through electrostatic interactions with phospholipid head groups [19], which further promotes drug leakage (Fig. 2K). This design correlates with the significant enrichment of calcium signaling pathways identified in KEGG analysis (Fig. 5C) and aligns with previous findings on piezoelectricity-induced Ca^2+^ overload [11]. Building upon this framework, we systematically optimized the composition of the hybrid core and overall physicochemical parameters. The CaCO_3_ to CMCS ratio was guided by established CMCS-mediated CaCO_3_ mineralization strategies and was iteratively optimized in our formulation to achieve uniform particle morphology, stable mineralization, efficient Toy loading, and pH-responsive degradation behavior [40]. Subsequently, considering injection flowability, dispersion stability, and preliminary in vivo efficacy, we determined that a particle size of approximately 200 to 250 nm and a surface zeta potential of −30 to −40 mV represented the optimal formulation parameters. Literature and theoretical models indicate that nanoparticles in this size range can effectively diffuse through extracellular matrix gaps while minimizing rapid leakage commonly associated with <50-nm particles, thus achieving an ideal balance between intratumoral retention and tissue penetration [41]. This design is consistent with the prevailing understanding of intratumoral delivery, where surface charge, hydrophilicity/hydrophobicity, and extracellular-matrix-binding affinity are often more influential than size alone in determining transport efficiency [42,43]. Notably, the TIR of HN-T/BT@Lip + US reached 54.10%, markedly surpassing those of single-component treatments such as Toy alone (TIR = 25.39%) and BT@Lip + US (TIR = 26.99%) (Fig. S4). Compared with structurally simpler piezoelectric platforms (e.g., UBTO@Gel + US, TIR = 23.33%) and the clinically used agent doxorubicin (TIR = 32.80%), our nanoplatform demonstrated a marked therapeutic advantage, highlighting its structure–function synergy and translational potential [13].

Mechanistically, our integrated multi-omics analysis revealed the pivotal role of intratumoral microbiota–metabolite interactions in regulating ferroptosis sensitivity and shaping the antitumor response. Upon HN-T/BT@Lip + US treatment, ferroptosis-associated suppressor genes were significantly down-regulated (Fig. 5A), and intracellular GSH levels were markedly depleted (Fig. 9E), suggesting enhanced susceptibility to ferroptotic cell death. In parallel, the same treatment profoundly altered the composition of the intratumoral microbiota (Fig. 8B to D). By correlating microbial and metabolic profiles, strong associations were observed between specific bacterial taxa and key metabolites (Fig. S7); for example, changes in the abundance of particular microbial species were linked to altered levels of TMAO and NAC. Among these metabolites, TMAO was selected for functional validation based on 2 key considerations. First, untargeted metabolomics identified TMAO as one of the most significantly dysregulated metabolites, exhibiting both a large fold change and robust statistical significance in the volcano plot and differential screening (Fig S8). Second, from a biochemical perspective, TMAO is a prototypical microbiota–host co-metabolite: its levels are co-regulated by microbiota-mediated precursor transformation and subsequent host enzymatic oxidation, thus serving as an integrated indicator of the interplay between intratumoral microbiota and host metabolic state [44]. Functional validation further demonstrated that these metabolites exacerbated LPO induced by the nanoplatform (Fig. 9H and I), supporting a causal contribution of microbiota-related metabolic perturbations to ferroptosis modulation during treatment. These results are consistent with previous studies, suggesting that TMAO enhances ferroptosis sensitivity through multiple convergent mechanisms, including increased ROS production, inhibition of the Nrf2–GSH/GPX4 antioxidant axis, disruption of iron homeostasis, suppression of SLC7A11, and exacerbation of LPO and iron-dependent oxidative injury [45,46].

In addition to demonstrating potent antitumor efficacy, we also confirmed the excellent biosafety profile of the platform (Fig. S5). H&E staining of major organs showed no detectable inflammation or tissue damage, and mouse body weight remained stable throughout treatment, indicating that intratumoral administration caused no appreciable systemic toxicity (Fig. 4C). The properties of each component further support this conclusion: the CaCO_3_–CMCS hybrid core rapidly decomposes into Ca^2+^ and CO_2_ under mildly acidic tumor microenvironment conditions and is a widely recognized biodegradable inorganic material [47]; liposomal components undergo enzymatic metabolism via endogenous lipases and phospholipases [48]; BT is a lead-free piezoelectric material with proven biocompatibility [49]; and Toy functions as an endoplasmic reticulum stress inducer that synergizes with the piezoelectric effect to amplify ROS-mediated cytotoxicity. Notably, intratumoral injection markedly limits nanoparticle entry into systemic circulation, thereby minimizing off-target exposure to major organs such as the liver and spleen [50].

In summary, we developed HN-T/BT@Lip, a US-activated nanoplatform capable of inducing ferroptosis and proposed a mechanistic model in which nanomaterials modulate the intratumoral microbiota–metabolism–ferroptosis axis to enhance therapeutic efficacy. Several limitations warrant consideration. As a mechanistic proof-of-concept study, further translational advancement will require continued efforts in process standardization, quality assurance/quality control, and delivery strategy optimization. Although multi-omics profiling is not yet widely implemented in routine clinical workflows, it remains valuable for delineating intratumoral microbiota–metabolism heterogeneity and identifying candidate regulatory signatures that may ultimately be refined into clinically actionable biomarkers. Because this study employed local intratumoral administration with a relatively short observation window to ensure controlled and reproducible intratumoral exposure, it was not designed to fully characterize systemic delivery, biodistribution, immune responses, or long-term biosafety. Future work will proceed in 3 directions: (a) dissecting the context-dependent mechanisms of ferroptosis regulation across distinct tumor microecological phenotypes through causal perturbation of specific microorganisms or metabolites, complemented by ferroptosis rescue experiments; (b) developing personalized ferroptosis-based therapeutic strategies guided by tumor microecological profiling; and (c) conducting rigorous long-term pharmacokinetic, immune-interaction, and chronic-toxicity evaluations to strengthen the evidence base for manufacturability, scalability, and translational feasibility of the platform.

## Ethical Approval

All experimental procedures were approved by the committee on the ethics of animal experiments of the Ethics Committee of the Medical College of Qingdao University (QDU-AEC-2024781). All animal housing and experiments were conducted in strict accordance with the institutional guidelines for the care and use of laboratory animals.

## Data Availability

The data supporting the findings of this study are available from the corresponding authors upon reasonable request. In addition, the authors confirm that the data supporting the findings of this study are available at PRJNA1331929 (NCBI).
