# Supplementary material for: Ultrasound-Activated Nanoplatform Counteracts Triple-Negative Breast Cancer via Remodeling Intratumoral Microbiota–Metabolism and Inducing Ferroptosis
Source: Biomater Res. 2026 Feb 3;30:0317. doi: 10.34133/bmr.0317 (PMC12864655; doi:10.34133/bmr.0317)
Supplement: Supplementary 1 — Tables S1 and S2 Figs. S1 to S8 [file bmr.0317.f1.docx]

**Supporting Information**

**Ultrasound-activated nanoplatform counteracts triple-negative breast cancer via remodeling intratumoral microbiota–metabolism and inducing ferroptosis**

Shuao Li^1†^, Yuxiu Gao^1†^, Danni Jiang^1†^, Xiaoyu Wu^2^, Yanan Feng^1^, Fang Chen^1^, Ningning He^2^, Shangyong Li^2,1^, Luxia Jing^1*^, Cheng Zhao^1*^

1. Department of Abdominal Ultrasound, The Affiliated Hospital of Qingdao University, Qingdao, Shandong, 266003, China

2. School of Basic Medicine, Qingdao Medical College, Qingdao University, Qingdao, 266071, China

^†^These authors have contributed equally to this work and share first authorship.

Address correspondence to:

qdfyzhaocheng@qdu.edu.cn (C.Z.); 20111210146@fudan.edu.cn (L.J.)

**Table S1.** EE and LC of Toy in HN-T NPs with different feed ratios.

| Toy:HN  (mass ratio) | EE(%) | LC(%) |
| --- | --- | --- |
| 1:1 | 24.40% | 20.33% |
| 1:2 | 32.25% | 16.04% |
| 1:3 | 33.82% | 10.57% |

**Table S2.** EE and LC of Toy in HN-T/BT@Lip with different feed ratios.

| DOPE:HSPC  (mass ratio) | EE(%) | LC(%) |
| --- | --- | --- |
| 3:5 | 47.67% | 5.45% |
| 1:1 | 44.39% | 5.22% |
| 5:3 | 37.85% | 4.70% |


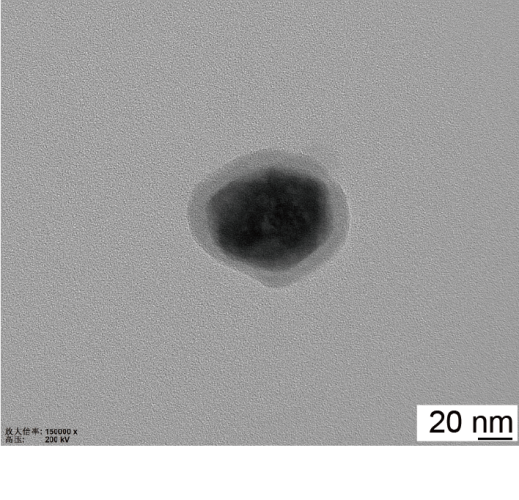


**Fig. S1.** TEM of HN-T (scale bar, 20 nm)


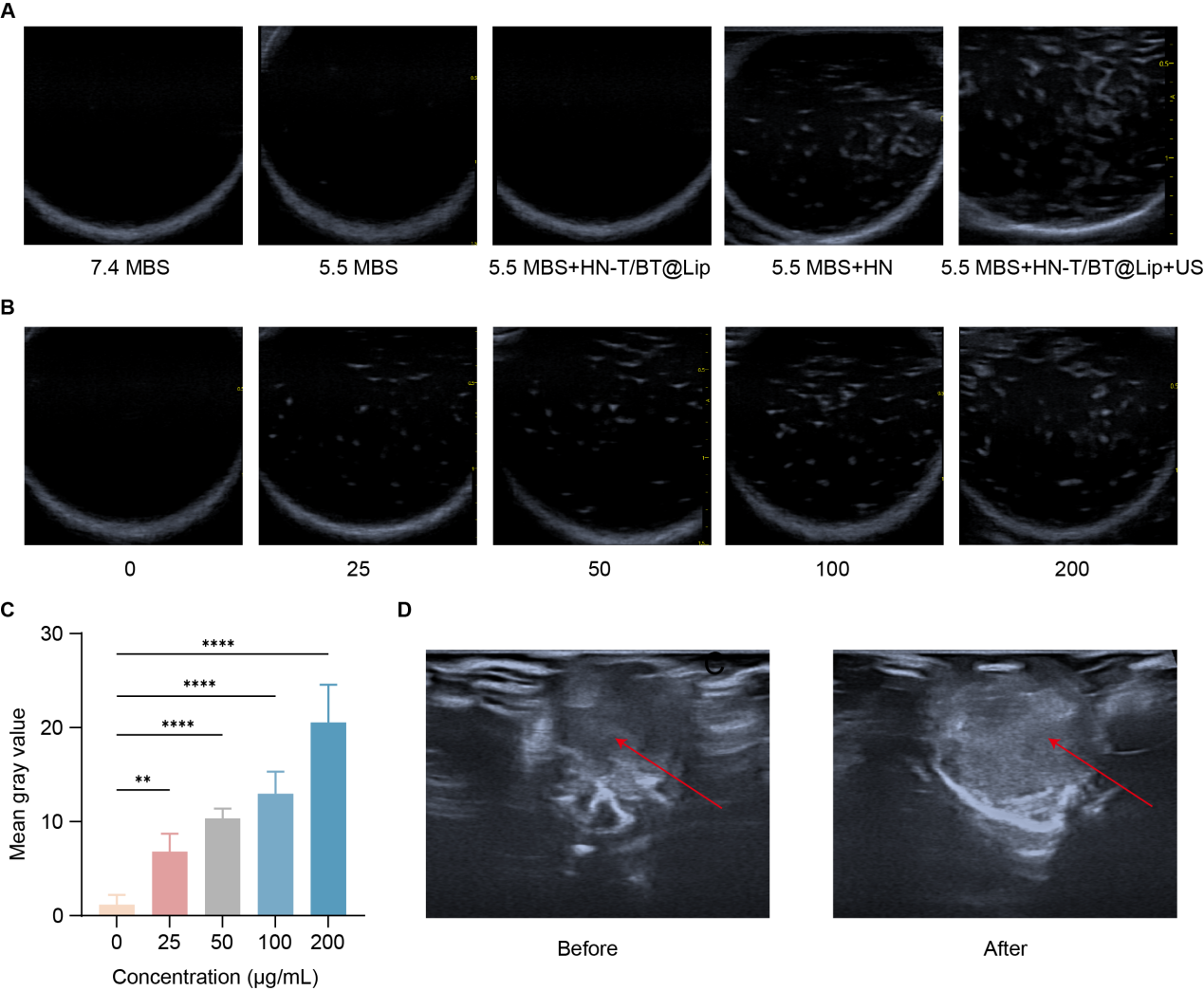


**Fig. S2. Ultrasound imaging performance of the nanoplatform.** (A) In vitro echogenicity of different formulations. (B) Concentration-dependent ultrasound images (0–200 μg/mL). (C) Quantification of mean gray values showing dose-dependent enhancement. (D) In vivo tumour ultrasound imaging before and after intratumoral injection of HN-T/BT@Lip.


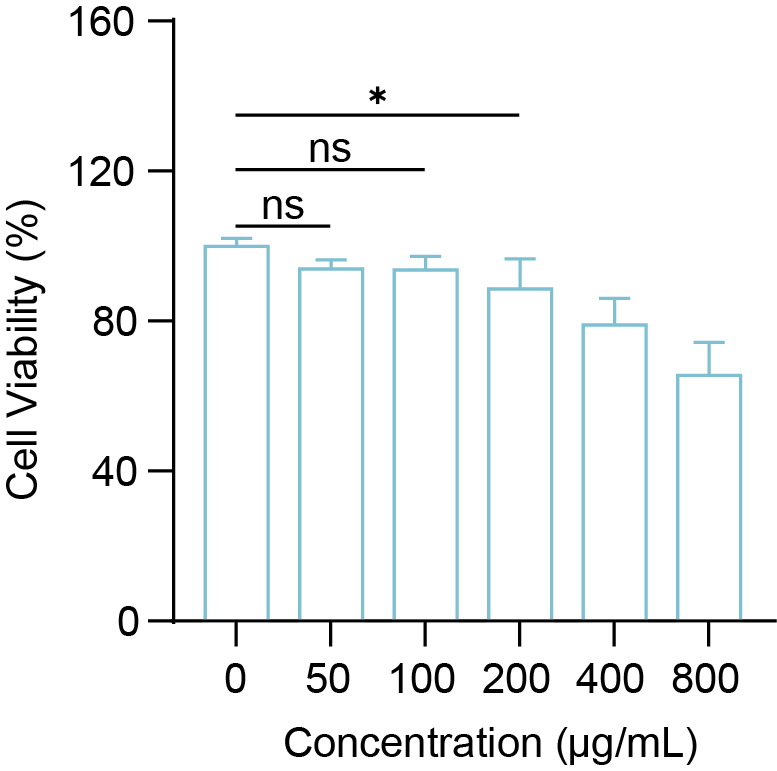


**Fig. S3.** Viability of 4T1 cells treated with BT@Lip.


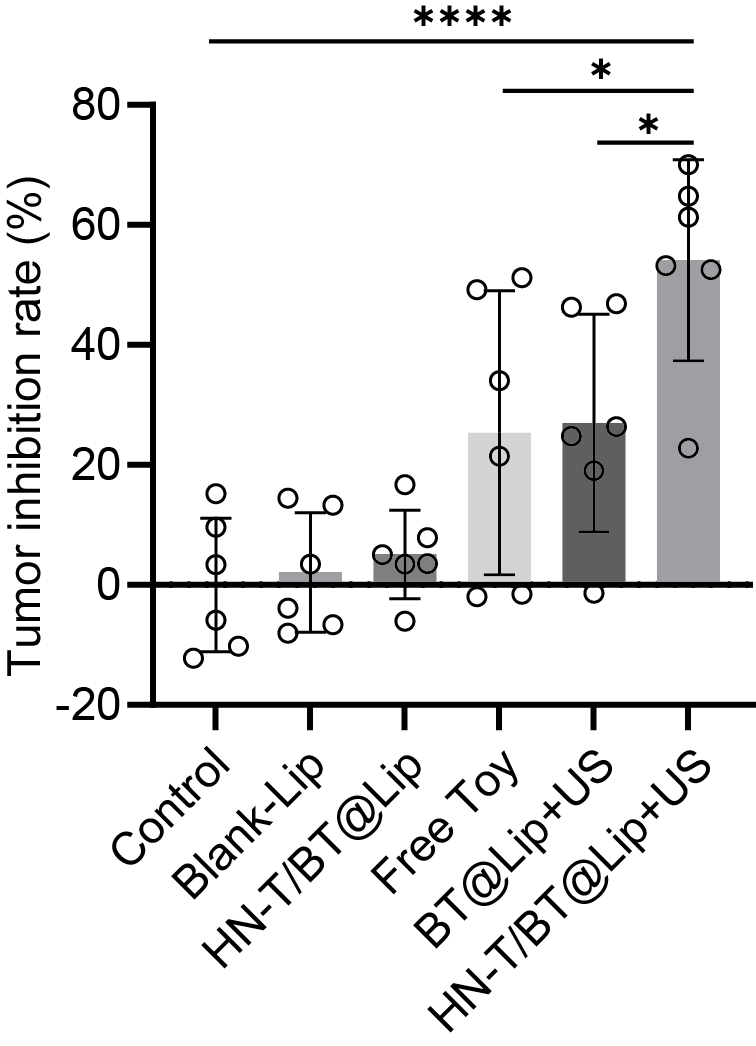


**Fig. S4.** Tumor inhibition rate (TIR) across different treatment groups.


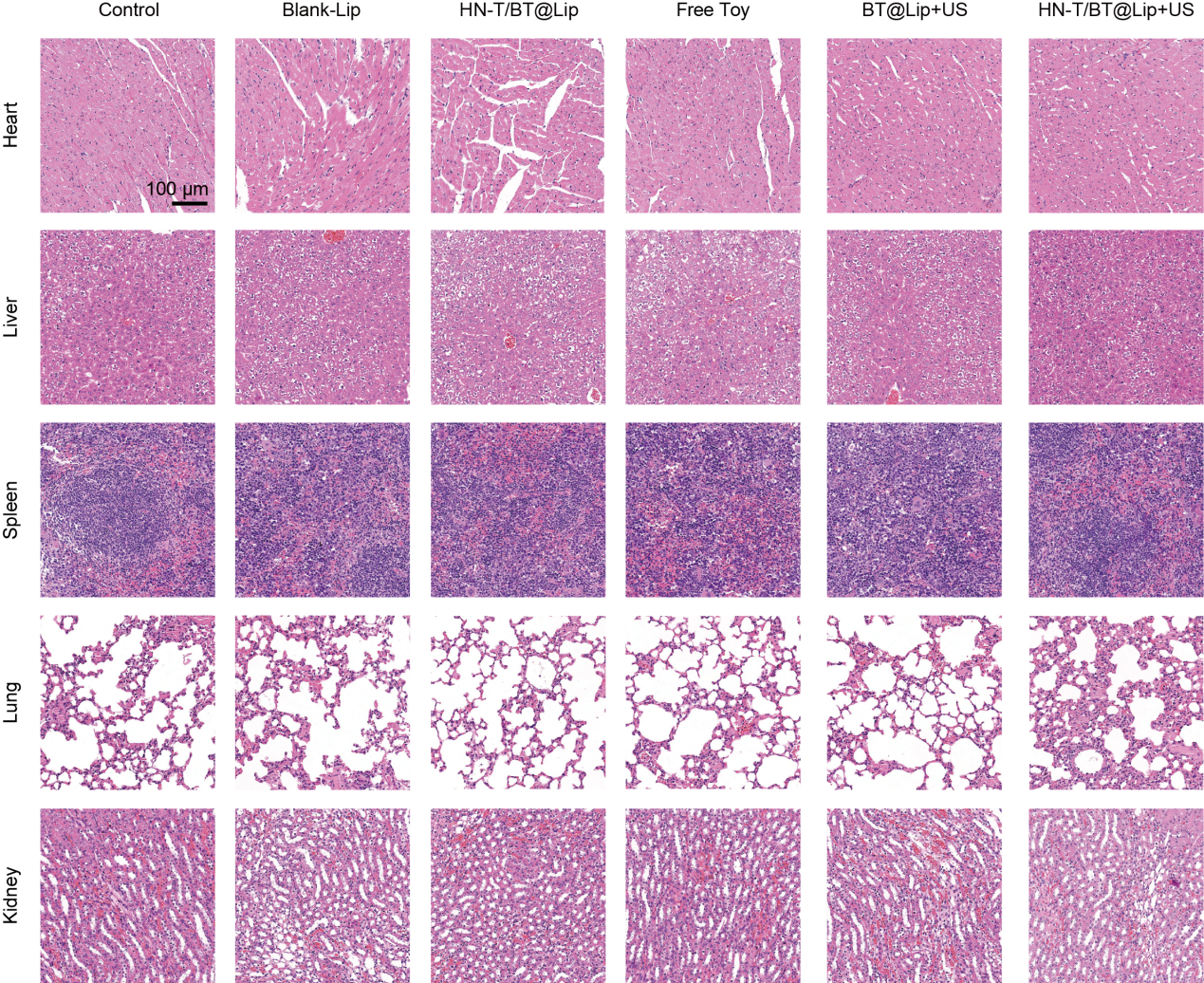


**Fig. S5.** Representative H&E-stained histological sections of major organs (heart, liver, spleen, lung, and kidney) from mice in different treatment groups (Control, Blank-Lip, HN-T/BT@Lip, Free Toy, BT@Lip + US, and HN-T/BT@Lip + US). Scale bar: 100 μm.


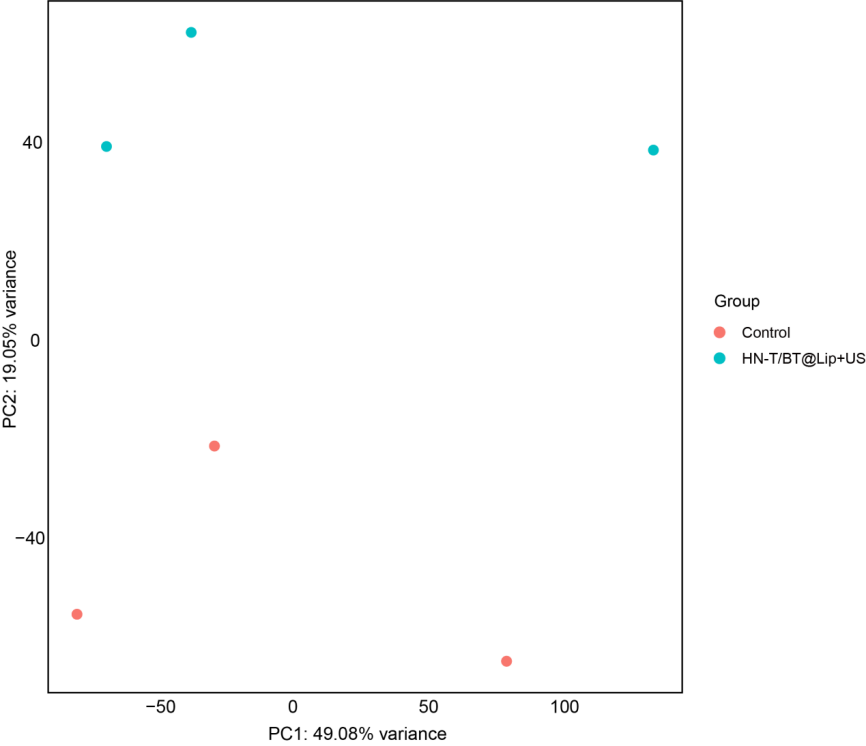


**Fig. S6.** PCA revealed a clear segregation between the HN-T/BT@Lip + US and Control groups.


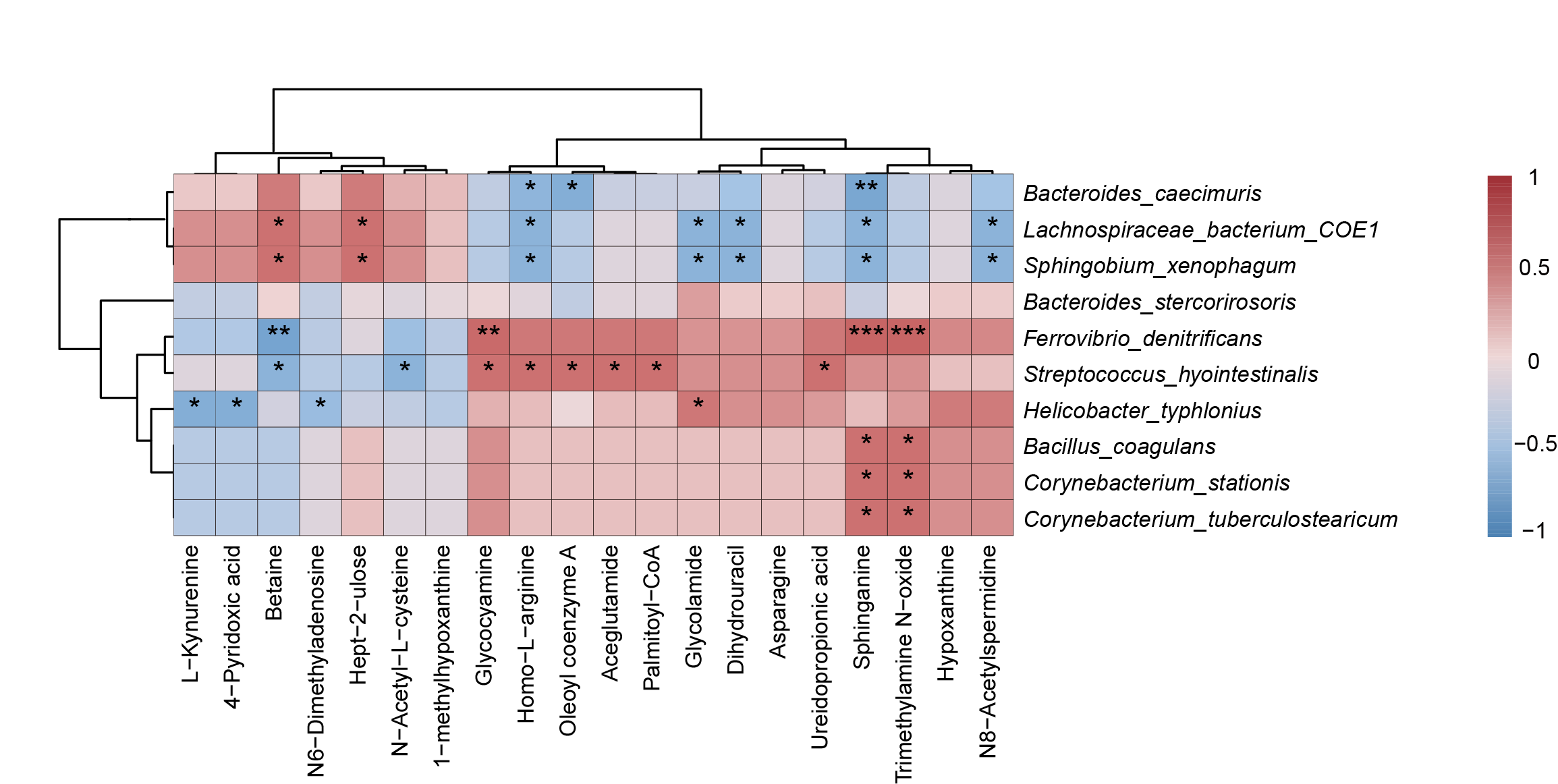


**Fig. S7.** Heatmap showing the correlations between representative microbial taxa and metabolites. The color scale represents Spearman correlation coefficients (red: positive; blue: negative).


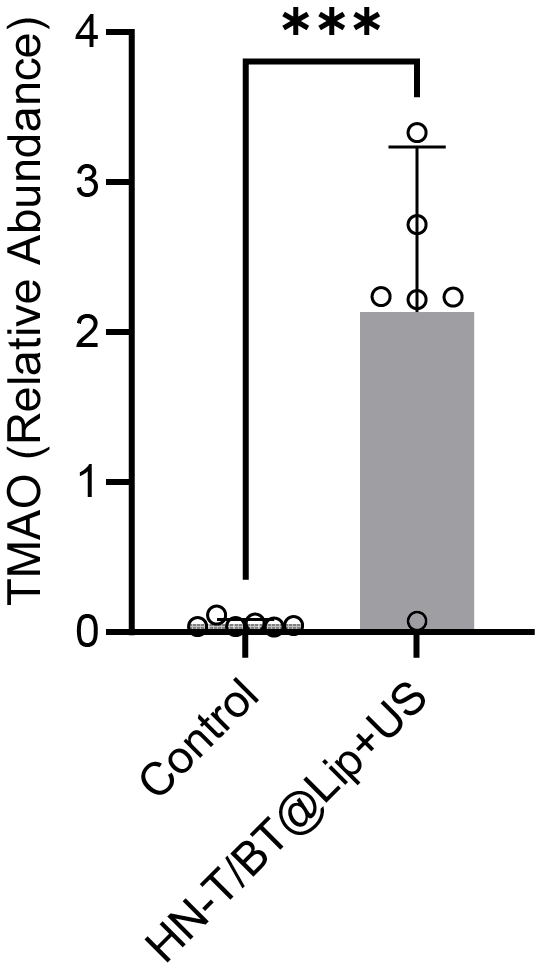


**Fig. S8.** Quantification of intratumoral TMAO levels showing a significant elevation following HN-T/BT@Lip + US treatment.
